# Division of labour in a matrix, rather than phagocytosis or endosymbiosis, as a route for the origin of eukaryotic cells

**DOI:** 10.1186/s13062-020-00260-9

**Published:** 2020-04-28

**Authors:** Andrew Bateman

**Affiliations:** 1 Division of Experimental Medicine, Department of Medicine, McGill University, Glen Site Pavilion E, 1001 Boulevard Decarie, Montreal, Quebec H4A 3J1 Canada; 2 Centre for Translational Biology, Research Institute of McGill University Health Centre, Glen Site Pavilion E, 1001 Boulevard Decarie, Montreal, Quebec H4A 3J1 Canada

**Keywords:** Eukaryotes, Eukaryogenesis, Evolution, Prokaryotes, Hypothesis, Matrix, Biofilm, Archaea, Bacteria, Mitochondria, Chromosomes, Membranes

## Abstract

**Abstract:**

Two apparently irreconcilable models dominate research into the origin of eukaryotes. In one model, amitochondrial proto-eukaryotes emerged autogenously from the last universal common ancestor of all cells. Proto-eukaryotes subsequently acquired mitochondrial progenitors by the phagocytic capture of bacteria. In the second model, two prokaryotes, probably an archaeon and a bacterial cell, engaged in prokaryotic endosymbiosis, with the species resident within the host becoming the mitochondrial progenitor. Both models have limitations. A search was therefore undertaken for alternative routes towards the origin of eukaryotic cells. The question was addressed by considering classes of potential pathways from prokaryotic to eukaryotic cells based on considerations of cellular topology. Among the solutions identified, one, called here the “third-space model”, has not been widely explored. A version is presented in which an extracellular space (the third-space), serves as a proxy cytoplasm for mixed populations of archaea and bacteria to “merge” as a transitionary complex without obligatory endosymbiosis or phagocytosis and to form a precursor cell. Incipient nuclei and mitochondria diverge by division of labour. The third-space model can accommodate the reorganization of prokaryote-like genomes to a more eukaryote-like genome structure. Nuclei with multiple chromosomes and mitosis emerge as a natural feature of the model. The model is compatible with the loss of archaeal lipid biochemistry while retaining archaeal genes and provides a route for the development of membranous organelles such as the Golgi apparatus and endoplasmic reticulum. Advantages, limitations and variations of the “third-space” models are discussed.

**Reviewers:**

This article was reviewed by Damien Devos, Buzz Baum and Michael Gray.

## Background

All known cells are either prokaryotic or eukaryotic. The prokaryotes encompass two domains, Bacteria and Archaea. The third domain, the Eukarya, differs from prokaryotes in ways that include larger cell size; intracellular partitioning into membrane-enveloped spaces, notably cytoplasm, nucleus and mitochondria, each supporting a subset of cellular functions (division of labour), spliceosome processing of mRNA, nucleoli, and the organization of the eukaryotic genome into multiple intron-rich linear chromosomes often with long intergenic spacer regions (Table 1). Recent estimates date the age of the first eukaryotic cells to between 2.1 [1] and < 1.84 Ga [2] years ago, making them very much younger than prokaryotes, and placing their emergence after the Great Oxidation Event [3] that enabled aerobic metabolism to become prevalent. Phylogenetic analyzes show that the last common ancestor of all eukaryotes exhibited most of the core molecular and functional characteristics of contemporary eukaryotic cells [4]. Understanding the origins of this structural and molecular complexity poses numerous problems [4–6].

**Table 1:** A summary of some of the key hallmarks that distinguish archaeal, bacterial and eukaryotic cells. Further details are presented in the text

Property	Archaea	Bacteria	Eukarya
Typical Size (diameter)	0.5-4 μm	0.5-4 μm	1.0-100 μm
Chromosome(s) and mRNA	Circular, compact, with polycistronic operons.	Circular, compact, with polycistronic operons.	Linear chromosomes, genes with introns, long intergenic sequences. Monocistronic transcripts, complex spliceosome, mRNA poly A tail. Nucleolus.
Internal structure	No membrane-enveloped organelles. No nucleus.	No membrane-enveloped organelles. No nucleus.	Membrane-enveloped organelles, mitochondria and nucleus.
Cytoarchitecture	simple	simple	complex
ATP-generation	Chemiosmosis across the cell membrane	Chemiosmosis across the cell membrane	Chemiosmosis across the mitochondrial membrane.
Membrane lipids	• Glycerol-ether lipids. • *sn*-glycerol-1-phosphate backbone. • Isoprenoid side chains.	• Glycerol-ester lipids. • *sn*-glycerol-3-phosphate backbone. • Fatty acid side chains.	• Glycerol-ester lipids. • *sn*-glycerol-3-phosphate backbone. • Fatty acid side chains.

**Fig. 1:**
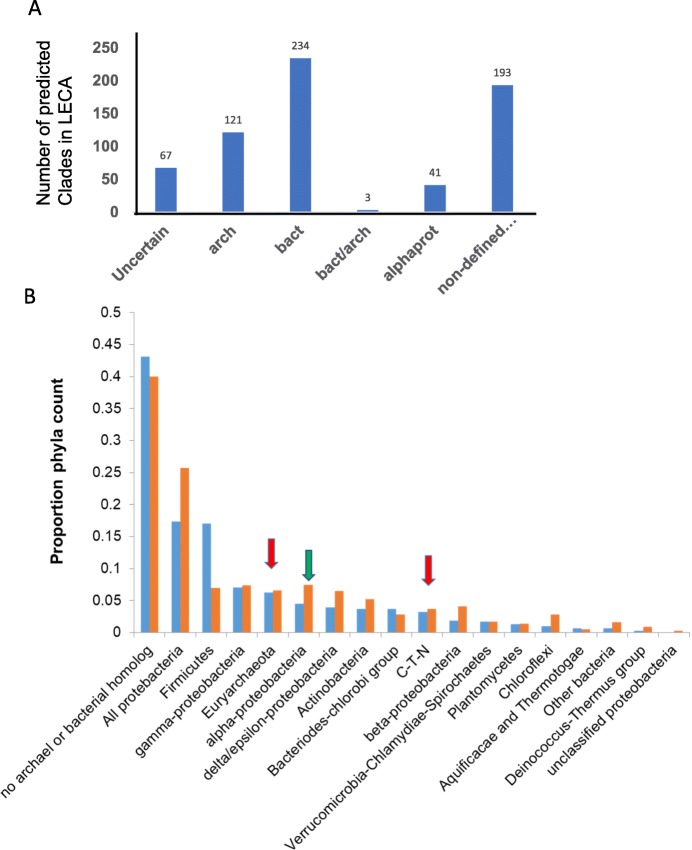
Prokaryotic origins of eukaryotic genes. (A) The phylogenetic distribution of gene clades from a proposed model of the Last Eukaryotic Common Ancestor (LECA). The data was taken from [13] in which 434 LECA clades were identified. Of these 67 were of “uncertain” origin in which Archaea and Bacteria appeared mixed, 121 were of archaeal origin, and 234 of bacterial origin. Among the bacterial clades, 41 were clearly alpha-proteobacterial (from the proposed precursors for mitochondria), but the majority of the bacterial signal (labelled “non-defined”), while definitively bacterial, could not be confidently assigned to a phylum. The total bacterial columns (bact) is the sum of alpha-proteobacterial and non-defined bacterial clades. Trees were generated for eukaryotic, bacterial and archaeal gene families. These were then analyzed in terms of “configurations”, for example, those that branched cleanly between eukaryote and bacteria, were assigned as bacterial clades, etc. Only 3 clades (labelled bact/arch) have the so-called “three domain configuration”, that branched between Archaea, Bacteria, and Eukarya with no obvious bias between the three domains. (B). The proposed prokaryotic origins of genes in two extant organisms, a yeast (*Saccharomyces cerevisiae*, blue bars) and a red alga (*Cyanidioschyzon merolae*, orange bars) are compared. The data was taken from [8], and redrawn to eliminate the contribution from cyanobacteria since, in red algae, many of these genes would have been acquired subsequent to eukaryogenesis. A data point has been added to include the genetic contribution ascribed to all proteobacteria combined, (alpha, beta, gamma, delta/epsilon and unclassified proteobacteria). Approximately 60% of eukaryotic genes are attributed to an origin among prokaryotic sources; of these approximately 10% have an archaeal background (red arrows) and 50% have a bacterial background. Note that, as in panel (A), bacterial sources outnumber archaeal sources, and that the genes derived from alpha-proteobacteria (green arrow), show no evidence of over-representation. C-T-N stands for Crenarchaeota-Thaumarchaeota-Nanoarchaeota

The prokaryotic affinities of many eukaryotic genes are well established [7–12] (Fig. 1). Eukaryotic genes that are of probable archaeal derivation often encode for information processing and translational proteins [11, 12]. For example, 67 of 78 eukaryotic ribosome proteins are also found in Archaea while only 34 are also found in Bacteria [14]. Archaeal-derived genes may, nevertheless, contribute to important non-informational processes [15–18], but, regardless of their function, mutations of archaeal-derived genes are more likely to exhibit a lethal phenotype in yeast than are mutations of bacterial-derived genes [9]. Correspondingly, genes of bacterial origin often encode for metabolic or operational proteins [11, 12]. This differentiation of function is not rigid, however, as many nuclear proteins have a bacterial origin [9, 19, 20], and a sub-group of eukaryotic genes display a chimeric bacterial/archaeal derivation, with the bacterial component dominant even among genes encoding for information processing proteins [20]. The origins of approximately 40% of eukaryotic genes are not easily assigned to either prokaryotic domain (Fig. 1).

It is widely [21–26], but not universally [27], agreed that mitochondria arose from Bacteria. Notably, mitochondria possess a set of non-nuclear genes that have clear phylogenetic affinities to corresponding alpha-proteobacterial genes [23–25]. Moreover, some alpha-proteobacteria display unusual intracytoplasmic membranes with striking resemblance to mitochondrial cristae [28–30]. In this view, mitochondria are living fossils, preserving in modern eukaryotic cells features of their ancient prokaryotic ancestors, although how mitochondrial progenitors were incorporated into the eukaryotic precursor has been contested. In autogenic scenarios of eukaryogenesis, eukaryotic cells arose as complex amitochondrial entities, the proto-eukaryotes. Proto-eukaryotes would be expected to have several hallmark features of eukaryotes, such as some form of nucleus, for example, and have been phagocytic, allowing for the capture of the bacterial progenitors of mitochondria [10, 31–34]. The nature or origin of proto-eukaryote cells are not well defined (see review [35]) and their subsequent history from their divergence from a population of the last universal common ancestors to the emergence of true eukaryotes is unclear. To date no organism has been identified that fulfills the criteria demanded of a proto-eukaryote since all amitochondrial eukaryotes that are known arose by loss of the organelle from ancestors that possessed mitochondria [23, 36]. Whether, lacking mitochondria, they would have been energetically viable, has been questioned [37, 38]. In other scenarios, the last universal common ancestors generate only prokaryotes, with eukaryotes emerging secondarily by prokaryotic endosymbiosis [39]. One partner (or small population of partner cells) resides within the other and eventually assumes a mitochondrial role as the prokaryotic host and passenger fuse to become, ultimately, the parent of all eukaryotes [40, 41]. Given the monophyly of extant eukaryotes, the formative endosymbiotic event is thought to have been rare, or even unique [8], and has, therefore, been called the “singular endosymbiotic event” [42]. Various versions of the endosymbiotic model have recently been critically reviewed [43]. Current versions of this scenario involve an archaeal host, implying that eukaryotes are part of a shared eukaryotic-archaeal lineage [44–49]. Phylogenetic analyses confirm a close affinity between eukaryotes and Archaea [11, 44–46, 48–52], a relationship that was bolstered by the recent discovery of the archaeal Lokiarchaeota (or Asgardian family) [15–18]. Not all studies, however, find that Lokiarchaean genomes “bridge the gap” between prokaryotes and eukaryotes [53–57]. To date, only one Asgardian species has been cultured, *Prometheoarchaeum syntrophicum*, which displays unusual arm-like or tentacular extensions, and requires at least two other microorganisms for its growth in culture (a *Halodesulfovibrio* and *Methanogenium* species) [58, 59]. It has an archaeal isoprenoid lipid composition and no intracellular organelle-like structures. Based on its properties, the discoverers propose a model for eukaryogenesis of “entangle–engulf–endogenize” (or E^3^) [59]. While syntrophy is widespread among prokaryotes [60], true prokaryotic endosymbiosis appears to be rare, with, at present, only one well-characterized example that consists of two bacterial species that are, in turn, embedded within a third partner, the specialized cells of an insect [61–63]. No example is presently known of mixed endosymbiosis between Archaea and Bacteria [32, 60, 64], even though this is a prerequisite for many current models of endosymbiotic eukaryogenesis. Future work on cultured Asgardian microorganisms may shed light on the problem of prokaryotic endosymbiosis.

Recovering the detailed relationships among very ancient genomes is profoundly challenging with many opportunities for artifacts and error to enter the phylogenetic trees. Nevertheless, some general conclusions can be made, among them that eukaryotic genomes are mosaics of bacterial-derived, archaeal-derived and eukaryotic-specific genes. Eukaryotic genes that originated from the postulated archaeal host are outnumbered by genes of bacterial origin (Fig. 1) [7, 8, 11, 13, 65]. Estimates for the relative bacterial to archaeal gene contribution vary from approximately 6 to 1 in representative unicellular organisms [8] to 2 to 1 in a phylogenetic reconstruction of the last eukaryotic common ancestor [13]. Overall, the alpha-proteobacterial progenitors of mitochondria contributed from around 6% [8] to 9.5% [13] of eukaryotic genes (Fig. 1), with between 51% [8] to 45% [13] of eukaryotic genes attributed to horizontal gene transfer from a highly mixed or taxonomically undefinable spectrum of bacteria other than alpha-proteobacteria. Correspondingly, only approximately 10% of the yeast mitochondrial proteome is alpha-proteobacterial in origin [66]. Horizontal gene transfer from Bacteria to Archaea had a major role in the evolution of some archaeal taxa [67–71], and, given the complex phylogenetic origin of eukaryotic genes (Fig. 1), appears to have played an even greater role in the origin of eukaryotes. Nevertheless, it is unclear why the founding cells of the prokaryotic endosymbiosis would cede precedence to a mixed and ill-defined population of secondary gene-donors to such an extent (90% in the case of the founding mitochondrial alpha-proteobacteria [66], and between 70 to 83% for the archaeal parents [8, 13]). Proteomic and protein fold analyses are not fully supportive of the standard prokaryotic endosymbiosis model [10, 72–74]. Current models of prokaryotic endosymbiosis propose that the nucleus originated as a response to the acquisition of introns [75, 76]. The intron hypothesis, however, provides limited insight into how the emerging eukaryotes traversed the immensely complex network of linked structural and functional transitions that must occur in lock-step for prokaryotic endosymbiotic partnerships to give rise to nucleated cells. The lipid composition of eukaryotic membranes differs fundamentally from archaeal cells and is much closer to that of bacterial membranes (Table 1, [77]). If the host cell of the ancestral endosymbiotic partnership was archaeal, as is often proposed [34, 64, 77], then, at some stage of eukaryogenesis, it must relinquish its characteristic archaeal membrane-lipid biosynthetic pathways in favour of those of the bacterial passenger cells. The mechanisms and evolutionary rationale underpinning this transition remain unclear.

Alternate hypotheses that confront some of these issues propose, for example, that the metabolic mosaicism of the mitochondria resulted from repeated nested endosymbioses, with one mitochondrial endosymbiont species being replaced by a second, and so on, over time [63]. Syntrophic cell-cell interactions have been suggested as the foundation of the prokaryote to eukaryote transition [78–80]. Syntrophy may, for instance, have led to sequential endosymbiosis, with a syntrophic archaeal cell taking residence within a bacterial partner to generate an endosymbiotic chimera with an archaeal nucleus [79] that was then followed by endosymbiosis of a syntrophic mitochondrial precursor cell [79]. In the “inside-out” topological model, mitochondrial progenitors living in syntrophic association with an archaeon were captured by cytoplasmic blebbing of the central archaeal cell that then served as the precursor for the nucleus [80].

Given that the prerequisite conditions for the autogenic and endosymbiotic narratives of eukaryogenesis have yet to be demonstrated, that is, the existence now, or in the past, of amitochondrial proto-eukaryotes whose ancestors never possessed mitochondria, or the presence of bacterial cells living in prokaryotic endosymbiosis within archaeal cells, alternative routes towards eukaryotic evolution were sought. Rather than employ phylogenetic or metabolic arguments the question was restated in terms of minimal cellular topologies. The eukaryotic cell is abstracted into its secondary spaces and the range of pathways is enumerated that lead from archaeal and bacterial cells, similarly abstracted, to the eukaryotic topology. These analyses provide frames of reference upon which putative eukaryogenic pathways can be rationally constructed. The biological plausibility of the pathways derived in this way is then evaluated by hypothetical step-wise reconstruction of the hallmark features of eukaryotic cells (as listed in Table 1), that is, by identifying possible biological correlates through which any novel pathway might account for the properties of eukaryotic cells. For simplicity, only archaeal and bacterial genome contributions are included in the analysis, ignoring other potential gene sources such as viruses [81], or hypothetical lineages that have not survived to the present (chronocytes) [33].

## Development of the third-space model of eukaryogenesis

Model development is broken into three stages. In the first section a theoretical analysis demonstrates the existence and logical coherence of novel potential frameworks for eukaryotic evolution. The second section (from “*Animating the strong third-space model*” to “*Steps towards a eukaryotic cytosol and mitochondria*”) investigates biological processes that, based on these new frameworks, would allow for the development of complex cells. The third section outlines the strengths and drawbacks of the model.

### Minimal cellular topologies

In the context of this manuscript a cellular space is considered as an abstract representation of the fundamental functional components of an idealized cell reduced to their most elemental depiction (the spatial division of labour). The spaces are nested as a hierarchy of primary, secondary and tertiary spaces. Primary and secondary spaces are typically delineated by membranes. Tertiary space may or may not be membrane bound (compare for example the lysosome and the nucleolus). In any given cell a space may contain one or many members (a multinucleated cell, for example, is still considered to have only one nuclear space although it has more than one nucleus). The representation allows simple questions to be asked about the relationship between spaces that can loosely be called topological, for example whether a given space can or cannot contain another space within it. This allows different models of major organizational transitions, such as eukaryogenesis, to be interpreted as belonging to one or other class of transition as defined by the topological relationships involved in the transition.

#### Primary, secondary and tertiary cellular spaces

1. The primary topology is the cell and all it contains.
2. The secondary topology reduces cells to secondary spaces or domains. Eukaryotic cells are tripartite with three well-defined secondary spaces; a membrane-delimited cytoplasm, membrane-delimited mitochondria, and a membrane-delimited nuclear space. Prokaryotes comprise a single membrane-enclosed secondary space and are therefore secondarily unipartitite. The identity of each space is defined by their dominant functions; RNA production (nucleus), metabolism and protein production (cytosol), metabolism and high-yield oxidative energy production (mitochondria). Individual spaces may be in physical communication (for example the cytosol and nucleus are connected via nuclear pores) and may share molecular components. The passage of molecules from one space to another is, however, selective, directional and regulated (gated)
3. Several eukaryotic groups have acquired additional secondary specialized spaces, for example, the chloroplasts of plants. This is outside the scope of this discussion.
4. The tertiary topology recognizes additional spaces that exist within the secondary spaces. These spaces are derived evolutionarily from the secondary spaces. The cytoplasm, for instance, is further divided into a series of tertiary spaces that include endoplasmic reticulum, Golgi apparatus, and vesicles. Similarly, the nucleolus is a tertiary space within the nucleus. In addition, some eukaryotes possess specialized barrier spaces such as the cell walls of plants and fungi. Tertiary spaces in prokaryotes include cell wall barrier spaces and the periplasm.
5. In eukaryotes, the nuclear space cannot contain the cytoplasmic or mitochondrial spaces. The mitochondrial space cannot contain the nuclear or cytoplasmic space. The cytoplasmic space contains both the nuclear and mitochondrial spaces and is, therefore, distinguished as the “containing space”.
6. Cellular topology is not a representation of the actual distribution and organization of functional elements inside a cell (the cellular topography). Each secondary space could represent from one to any number of individual elements. For example, mitochondria are represented topologically as a single space even though any given cell possesses numerous mitochondria.
7. Spaces can change their nature through evolution, for example a bacterial space can evolve into a mitochondrial space.

As an analogy, if the primary space is thought of as a continent, the secondary spaces represent countries that may be in contact with each other, and share goods, but maintain their own border posts and custom controls. The tertiary spaces are equivalent to districts within each country.

#### Three distinct pathways that may lead to the tripartite eukaryotic topology

**Fig. 2:**
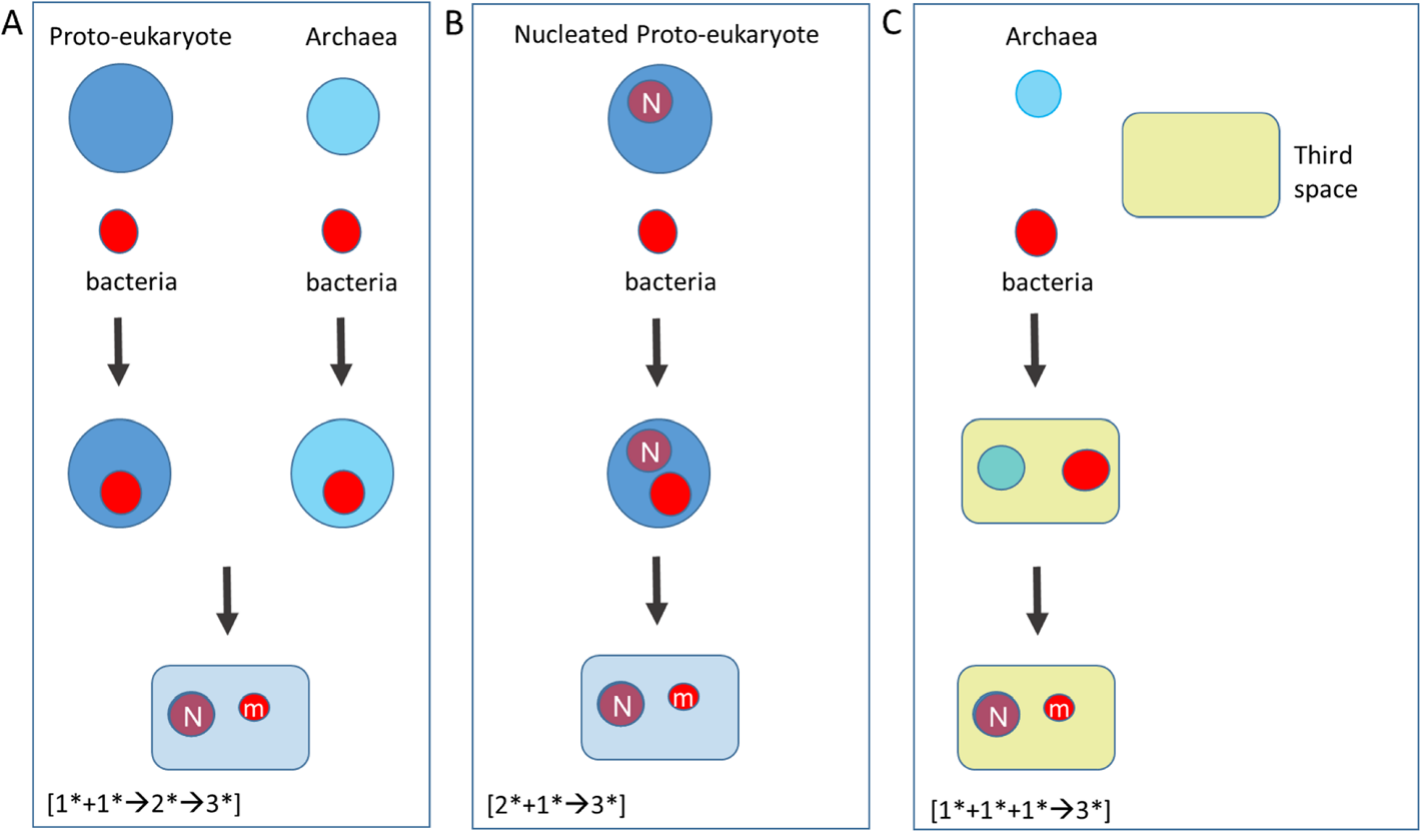
Three potential pathways from prokaryotes to eukaryotes. In (**a**) two unipartite spaces fuse to form a bipartitite space that develops into a tripartitite eukaryotic progenitor [1* + 1*➔ 2*➔ 3*]. The initial unipartite spaces represented here are either a non-nucleated autogenously-derived proto-eukaryote and a bacterial space, or an archaeal space and bacterial space. The bacterial space is assumed to be the progenitor of the mitochondrial space (m), and fusion of the partner genomes is assumed to give rise to the nuclear space (N). In (**b**) a bipartite space, assumed here to be an autogenously-derived nucleated protoeukaryote, merges with a unipartite space, assumed here to be bacterial rather than archaeal, [2* + 1* ➔ 3*] to generate a eukaryotic progenitor cell. In (**c**), three unipartite spaces merge to give a tripartite space, [1* + 1* + 1* ➔ 3*]. In this manuscript the three spaces are assumed to be an archaeal space, a bacterial space and a matrix or third-space. The matrix serves as a proxy-cytoplasm, the two cellular spaces both contribute to the nucleus

Given that the eukaryotic cell is a mosaic with archael and bacterial elements the minimum starting point is taken as two distinct primary spaces (one representing the bacterial space and one the archaeal space). Figure 2 presents potential pathways from prokaryotes or proto-eukaryotic cells to eukaryotic cells.

In Fig. 2a two unipartitite topologies (1* + 1*) merge to form a bipartitite (2*) topology that rearranges to form a tripartite topology (3*). This can be represented in a shorthand notation as [1* + 1*➔2*➔3*], where * represents a primary cellular topology and the number indicates how many secondary cellular topologies occupy the primary space. When applied to prokaryotic endosysmbiosis, the containing space is provided by the archaeon, and the final nuclear space arises de novo from the interaction of the archaeal and bacterial spaces. The [1* + 1*➔2*➔3*], topology applies also to the fusion of an autogenously-derived non-nucleated proto-eukaryote with mitochondrial precursor cells, where the containing space is provided by the amitochondrial protoeukaryote. In the “inside-out” model the archaeon provides both the containing space and the nuclear space which must bifurcate into two distinct spaces (nucleus and the containing cytosol) at the 2*➔3* transition.

In Fig. 2b a bipartite space and a unipartite space give rise to a tripartite space [2* + 1*➔3*]. This might represent, for example, an autogenously-derived nucleated but amitochondrial proto-eukaryote (cytoplasm + nucleus) merging with a unipartite space (mitochondrial progenitors) from which a tripartite space emerges. 2A and 2B can be combined ([1* + 1*➔2*; 2* + 1*➔3*]).

**Fig. 3:**
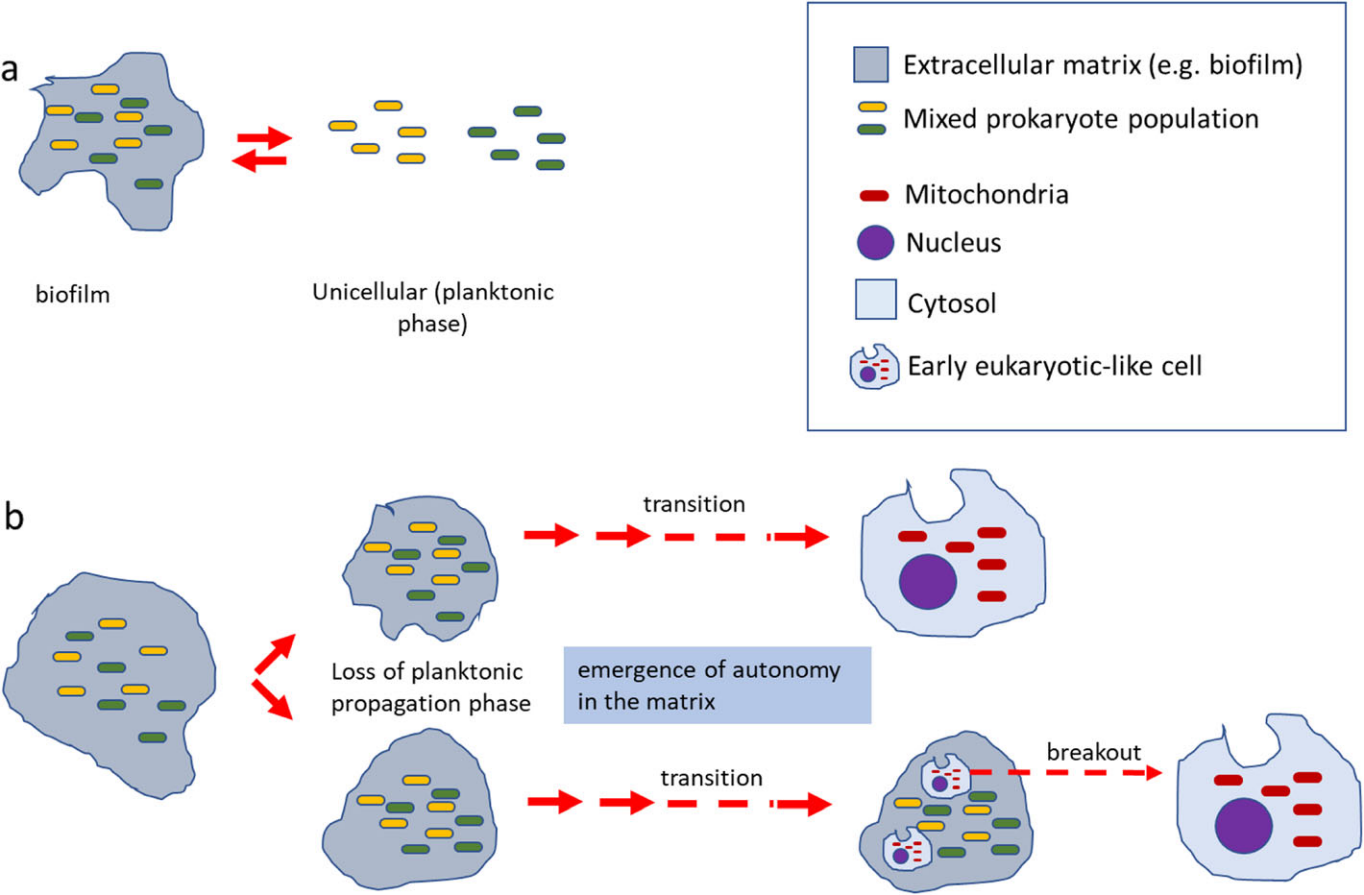
Cell-like emergence from a matrix ensemble. **a** A consortium of cells in a matrix propagates by matrix dissolution to release cells as a unicellular planktonic phase cells that disperses and may reform a biofilm. **b** A stable matrix/cell ensemble undergoes progressive transitions towards an autonomous cell-like life style. Either the entire ensemble evolves into a cell-like life form (upper pathway), or subsets of cells within the ensemble matrix progressively become sufficiently autonomous to survive without the support of the matrix (breakout, lower pathway)

In Fig. 2c, three unipartite spaces merge to form a tripartite topology [1* + 1* + 1*➔3*]. Following note 5 above, one space, called the third-space in Fig. 2c, should contain the other two spaces, providing a matrix for their reorganization into the specialized nuclear and mitochondrial spaces. In effect, the third-space exhibits the same topology as eukaryotic cytoplasm and serves as an incipient or proxy cytoplasm. Note that the [1* + 1* + 1*➔3*] third-space pathway does not correspond to either the autogenous or prokaryotic endosymbiosis models. An ensemble of cells co-evolving within a stable third-space (or matrix) (Fig. 2c) may contain sub-ensembles with different organizations. This can be represented as; [(1* + 1* + 1*): (1* + 1* + 1*➔2* + 1*): (1* + 1* + 1*➔3*)]. Here the third-space contains a mixed population of unipartite (1*), bipartite (2*) and tripartite (3*) sub-ensembles where the 3* sub-ensembles remain within the matrix. The possibility exists, however, for a 3* ensemble to escape or breakout of the matrix and assume an independent cell-like identity (Fig. 3). Some possible variations on the basic [1* + 1* + 1*➔3*] third-space transition are discussed below (see “Variations of the model”).

In its simplest version, the third-space model requires only two cell types, or a population of two cell-types, embedded in the matrix, one of which must be archaeal and the other bacterial. The model may also incorporate diversified populations composed of more than two cell types (all the Archaea are still considered as one space and all Bacteria as a second space Fig. 2).

#### Strong, weak and intermediate third-space models

Figure 2 identifies classes of solution for the problem of eukaryogenesis. The class of solutions summarized in Fig. 2a, for example, includes at least two distinct biological pathways. Similarly, the class of solutions summarized as the third-space model (Fig. 2c) may contain multiple pathways. These will be considered in terms of strong, intermediate and weak third-space models.

Prokaryotic endosymbiosis and autogeny both presume an intermediate bipartite stage (2*) in eukaryotic evolution (Fig. 2a and b). The strong third-space solution (Fig. 2c) eliminates the bipartite intermediate and presumes that the three distinct secondary spaces in the tripartite eukaryotic cell arose by the transformations of three distinct initial evolutionary substrates.

Intermediate third-space models are more complex and invoke a bipartite intermediate. In one version two primary spaces give rise to a matrix-embedded amitochondrial proto-eukaryote, (cytoplasm plus nucleus). Subsequent interaction allows to acquire the mitochondrial progenitors from additional cells embedded in the matrix, by, for example, phagocytosis. (This can be summarized as (1* + 1* + 1*➔2* + 1*➔3*). The intermediate third-space model thereby provides a potential route from simpler cells to a more complex proto-eukaryote, that side-steps a drawback of the autogenous proto-eukaryote model. Rather than surfacing from the last common universal ancestor along with prokaryotes, the formation of the proto-eukaryotes is now delayed until after the emergence of prokaryotes.

In weak third-space models, two spaces form a bipartite relationship within the matrix, but undergo the transition to the final tripartite state with no further cellular contribution from the matrix. An example would be the fusion of two cells in an endosymbiotic relationship (as in Fig. 2a) but inside a protective matrix. Other cells in the matrix provide a genomic reservoir to support extensive horizontal gene transfer. Since not all prokaryotic species will co-exist in equilibrium within a biofilm or similar matrix, the presence of the third-space effectively pre-selects or filters interaction between those species that can exist together in a cooperative partnership. This is a simple modification of the prokaryotic endosymbiosis model of eukaryogenesis, requiring only that the matrix facilitates the formation, survival and development of the endosymbiotic pair.

In summary, the underlying structural logic of eukaryogenesis predicts a novel class of frameworks for the origins of complex cellular structures that differs from either autogenic or endosymbiotic hypotheses of eukaryogenesis. There are variations of the model, namely weak, intermediate and strong, each starting either with two component populations or more complex mixed populations. Figure 2 does not, however, identify any biological steps that, when applied to this framework, would lead to the assembly of transitionary life forms capable of becoming primitive eukaryotic cells. The subsequent discussion is focused on the strong third-space model (Fig. 2c) with a mixed population and how it might provide for the emergence of eukaryotic cells.

### Animating the strong third-space model

#### What are the three spaces?

Given that genes of archaeal and bacterial origin occur in eukaryotes, the starting conditions are taken to include an archaeal space (which may include the recently discovered *Lokiarchaeota*) and a bacterial space. The third-space must: (i) Enclose the other spaces and be biologically compatible with them (see note 5 above in *minimal cellular topologies*). (ii) It must be sufficiently stable to provide the opportunity for eukaryotic-like properties to emerge over time. Stability may be provided either by long-term physical survival of the matrix and its cellular consortium, and/or by the ensemble acquiring the ability to efficiently regenerate after dispersal. This is addressed further in the section *"Limitations"*. (iii) It should be more than a passive arena for the evolution of eukaryotic cells but should be evolutionarily dynamic. As the third space evolves from a matrix to a cytosol it will have to pass through a series of transitionary phases that combine, to varying degrees, the properties of the matrix and the cytosol. These provides an increasingly privileged microenvironment that physically supports and protects the ensemble, but also serves as a locus for functional networks of increasing complexity that tie together the cellular elements of the ensemble as a coordinated system.

Geological micro-environments could provide protective and stable spaces [82] fulfilling criteria (i) and (ii), above, but it is unclear whether they could fulfill criterion (iii). Potential organic third-spaces include lipid droplets or organic extracellular matrices. Given the physical instability of lipid droplets, a stable extracellular matrix-like structure is of more interest. A cellular third-space either in a form that no longer exists, such as a chronocyte [33], or a prokaryote host, is a further possibility, but will only be discussed briefly (section *Cellular third spaces*) as there are no examples among living entities from which to draw inferences.

#### Extracellular matrices as models for the third-space

Most prokaryotes grow embedded in extracellular matrices in the form of biofilms or microbial mats [83], a mode of living that has been documented from rocks 3 to 3.5 Ga old [84]. Biofilms can support mixed populations of diverse species [85–87]. They are composed of polysaccharide polymers, proteins (including enzymes), extracellular DNA (eDNA), water, minerals and lipids, may be adherent or floating (flocs) and may vary in size from very large aggregates to microcolonies [85–88]. Smaller communities seem more likely to be most suitable as they are closer in size to the final eukaryotic cell. Cells within biofilms communicate by quorum sensing [89] and social networks can evolve that exhibit cooperation [90–92], altruism [93], the use of shared resources (public goods) [94], social cheating [95], and competition [96–98]. Division of labour can occur among cells in the matrix [99]. The biofilm is structured, possessing chemical gradients (aerobic to anaerobic for example [100]) and channels for the movement of nutrients [85–87]. Roles for biofilm matrices in eukaryogenesis have been proposed previously [101, 102], although the mechanisms invoked differ from those discussed here. Jeckely [101] proposed that early eukaryotic evolution occurred in a social biofilm-like context, with a sub-set of cells in the community behaving as social cheaters. These cells evolved into predators, first by lysis of other cells and ultimately by the acquisition of a eukaryote-like phagotrophic mechanism. Phagocytosis allows the subsequent uptake and incorporation of mitochondrial precursors as endosymbionts. Norris and Root-Bernstein [102] suggest that eukaryotes evolved from a mutualistic prokaryotic social structure such as a mat, biofilm or colony that underwent the exchange of “hyperstructures” (essentially pre-existing functional modules) between multiple cell types. This was achieved by the physical merger of the partner cells into a “meta-cell” that then underwent a process of integration and compaction or streamlining of structures to generate an entity more closely resembling a eukaryote.

Given the multiplicity of cells resident within biofilm-like matrices, and since “each secondary space could represent from one to any number of individual elements” (note 6, *minimal cellular topologies*, above), a biofilm-like third-space is compatible with the evolution of Eukarya from cell populations, possibly of mixed cell type. As noted above, the biofilm-like matrix is the initial condition for the third space but is structurally and functionally modified as eukaryogenesis proceeds (i.e. Matrix ➔ Transitionary phases ➔ Cytosol). Biofilms are example of a matrix that can be used to model the properties of the third-space, but it does not follow that there is third space has a one-to-one identity between any extant biofilm and the third-space matrix and other ther parameters, such as cell-cell aggregation may also play a role in organizing the three spaces of the model.

#### Allowable assumptions

Given the structure of the eukaryotic cell and the prokaryotic phylogenetic signature still evident in its nuclear, mitochondrial and chloroplast genomes, certain events can be proposed in the development of eukaryotic cells.

1. *Loss of cell walls.* Any model of eukaryogenesis that begins from prokaryotic cells has to accommodate the loss of the prokaryotic cell wall. Mitochondria and most chloroplasts have lost their cell walls. Examples of Archaea and Bacteria without cell walls are well known, for example, *Thermoplasma* and *Rickettsia*. Moreover, in an appropriately supportive environment, bacteria that normally fabricate cell walls nevertheless remain viable without a wall as so-called L-form cells [103, 104]. Cell division of L-form cells differs from equivalent species with intact cell walls. It is, for example, independent of the bacterial tubulin-homolog FtsZ [104, 105], and, at least in some instances, results in polypoid cells connected by lipid filaments capable of transferring cytoplasmic contents from cell to cell [105]. (FtsZ-like molecules are present in some, but not all, eukaryotes [106]).
2. *Cell Fusion.* Mitochondria are not discrete entities but instead often fuse to form dynamic tubular and network structures [107–109]. They are the most prokaryote-like of the three secondary spaces in eukaryote cells. It is reasonable, therefore, that similar fusion events could occur between the participating cells resident within the third-space matrix at least during some stages of the transition from prokaryotes to eukaryotes. This may be related to the loss of cell walls as above.
3. *Centralization of the genetic information.* Although mitochondria and chloroplasts have retained some genetic information, the genes for most of their proteins have been centralized within the nuclear genome. As this occurred independently for mitochondria and chloroplasts it is reasonable to assume that genome centralization is advantageous and that, during the transition from prokaryotes to eukaryotes, genetic information, from whatever source, will become concentrated into a centralized genome.
4. *Intron invasion.* The last common ancestor of eukaryotes possessed introns [110] probably derived from bacterial group II catalytic introns [75, 76, 79, 110–114]. These are self-splicing mobile elements that occur in the genomes of bacteria, mitochondria and chloroplasts [113, 115]. They are rare in archaeal genomes [116, 117]. Endosymbiotic models of eukaryogenesis propose that group II catalytic mobile elements from the endosymbiotic passenger cells “invaded” the genome of the host cell, although what specifically drove intron invasion and proliferation is not clear [118–121]. Whenever type II introns insert into coding regions, a conflict arises between the faster speed of ribosomal entry and read-through versus the slower rate of out-splicing of introns from the transcript [75, 76]. This results in the accumulation of nonsense transcripts. Moreover, with time, mutations would cause many “invader” type II elements to lose the ability to self-splice [114]. The genetic crises that ensued were resolved by the development of stand-alone spliceosome complexes [75, 76, 114] as well as the separation of RNA splicing and translation between the nucleus and cytosol respectively [75, 76, 79]. Other models of intron evolution are reviewed in [122].
5. *Chromosome linearization.* Although mitochondrial DNA is typically considered to be circular, linear mitochondrial genomes have evolved multiple times independently which implies a relatively low barrier between circular and simple linear chromosomes, allowing for an evolutionarily intermediate linear state between circular chromosomes and eukaryote-like centromeric/telomeric linear chromosomes [123, 124]. Telomerases are evolutionarily related to Type II catalytic introns [115] suggesting a role for these elements in supporting eukaryotic chromosome linearization.

### Putative steps towards nucleus formation in a third-space model

#### Genomic reorganization towards a eukaryotic pattern

The eukaryotic genome arose by merger of archaeal and bacterial genomes. The third-space model can accommodate simple populations of only two cell types (an archaeon and an alpha-proteobacteria) but also more complex mixed populations with more than two cell types. Considering mixed populations, and focusing on the bacterial component, the relative weakness of the alpha-proteobacterial signal (the mitochondrial precursor) compared to the aggregate bacterial signal in the eukaryotic nuclear genome (Fig. 1) can be rationalized if the population contained more than one type of bacteria (an alpha-proteobacterium plus others). A mixed population model does not, however, necessarily require the involvement of more than one archaeal cell type. The minimal mixed-population third-space model suggests, therefore, more than one type of bacterial cell interacting with one archaeal cell type (presumably one of the Lokiarchaeota).

Horizontal transfer of genes between prokaryotes is a fundamental property of their evolution, and has allowed for very extensive genetic mosaicism to occur in some cases, including in inter-domain mosaicism, where both archaeal and bacterial genes are prominent in the same organism [69]. For example, the evolution of archaeal *Halobacteria* was marked by the acquisition of approximately1000 bacterial genes families [67]. Interdomain mosaicism is similarly evident among *Lokiarchaea* species, 33% of their genes are unique to these species, with the remaining 36% of archaeal origin, and 28% bacterial [125]. Bacteria may likewise carry a significant archaeal genetic content, thus in *Thermotoga maritima,* 24% of genes have been imported from archaeal species. Horizontal gene transfer is promoted by growth in biofilm matrices, as has been demonstrated both in Archaea, in *Haloferax volcanii* [126], for example, and in Bacteria [127–129].

Biofilms deploy large amounts of DNA to the matrix (extracellular or eDNA) where it serves an essential structural role [130]. The reintegration of fragments of eDNA back into cellular genomes facilitates the horizontal transfer of genes between cells in the matrix [127, 128, 130–133]. Growth in close quarters confined in the matrix also favours gene exchange by conjugation [128, 129, 134–136]. In a population of cells living close to each other for extended periods within a stable matrix, and with their DNA cycling between genomic and extracellular phases, gene transfer will result in the progressive generation of cells carrying increasingly merged genomes. The recurrent mixing of genomes in a stable matrix would, over time, lead to dominance in the matrix of those merged genomes that confer selectively favoured phenotypes. Some innovations would be selected solely by virtue of their ability to enhance the survival of an individual cell type in the matrix (cis-selection), while others could, in addition, bestow benefits across multiple cell-types in the ensemble (trans-selection), perhaps by conferring matrix stabilization, or other improvements to ensemble functionality.

The transfer of genes can occur in either direction between Archaea to Bacteria [67, 69, 137]. Given the preponderance in eukaryote genomes of bacterial-like genes over archaeal-like genes (Fig. 1), and assuming a mixed-population model, it is probable that Bacteria outnumbered Archaea in the primitive matrix, favouring a net “bacterialization” of the archaeal component. In the well-established large-scale horizontal transfer of genes that occurred during the evolution of several archaeal lineages [67–69, 125], gene transfer is estimated to have occurred to approximately 5 fold greater extent from Bacteria to Archaea than in the reverse direction [68, 125]. The resemblance between the bacterial mosaicism of archaeal lineages and the merging of bacterial and archaeal genomes during their “eukaryogenesis” has been noted by others [70] and may reflect similar evolutionary processes, but with different evolutionary endpoints.

**Fig. 4:**
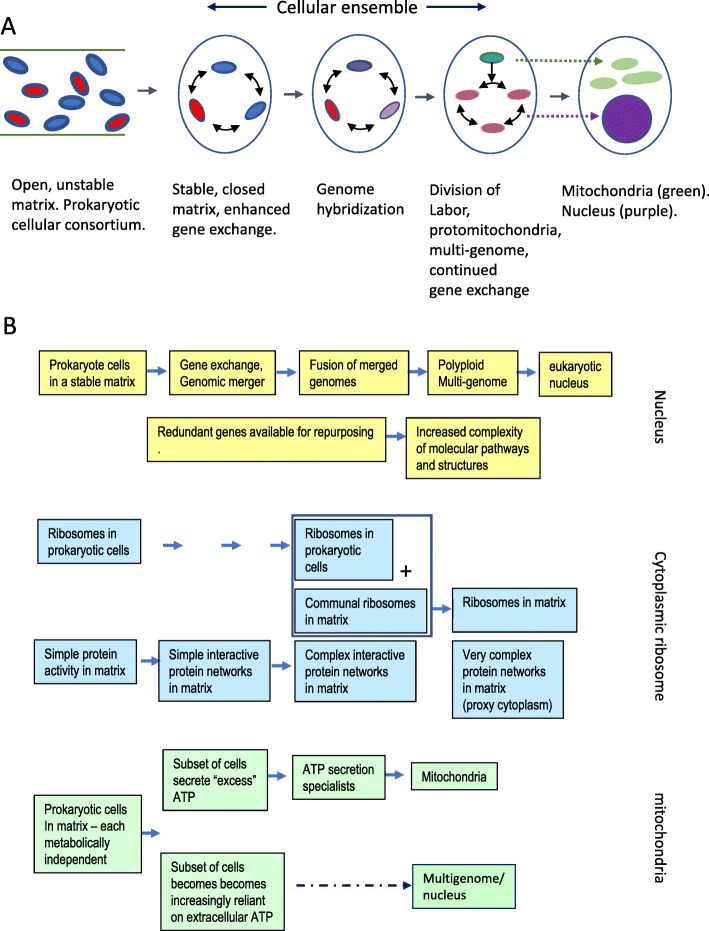
A brief outline of steps from prokaryotic cells in a matrix to a progenitor eukaryotic cell. **a** A transient consortium of prokaryotic cells (red Archaea, blue bacteria) in a biofilm-like or matrix undergoes a transformation to a stable ensemble of cells that encourages gene transfer resulting in increasingly hybridized genomes. Physical merger of cells results in multigenomes, with division of labour resulting in differentiation into mitochondria and nucleus. **b** In the yellow stream, a primitive nucleus emerges from a population of prokaryotic cells in a stable matrix through a succession of gene transfers resulting in merged genomes followed by cell fusions to result in multi-genome structures with multiple non-identical chromosomes. The genetic redundancy of the process supports the evolution of complex molecular pathways. **b** In the blue stream, developing interdependence among cells in a stable matrix allows for the emergence of increasingly elaborate protein networks that are supported by matrix-based (and ultimately cytoplasmic) ribosomes. In the green stream, as the population becomes progressively interdependent some cells in the matrix become ATP secretors, supplementing the energy needs of other cells and ultimately evolving into primitive mitochondria. Please consult the main text for more information

Although large-scale horizontal gene transfer did occur between Bacteria and Archaea, there seems to be no example of horizontal gene transfer alone transforming an archaeal or bacterial species into anything other than modified Archaea or modified Bacteria. Something else is therefore required, in addition to horizontal gene transfer, to achieve eukaryogenesis. Prokaryotic endosymbiosis has often been proposed as one such driver, but given its apparent rarity in nature, other possibilities should be explored. Any model of eukaryogenesis that incorporates prokaryotic progenitors requires, at some stage, the loss of prokaryotic cell walls. As the ensemble transitions towards an increasingly integrated life style, and with a sub-population of wall-free L-form cells (allowable assumption 1, *loss of cell walls*), the physical fusion of the wall-less hybrid genomes into increasingly larger structures, approaching something more like eukaryotic nuclei, is allowable under assumption 2 (*cell fusion*) and assumption 3 (*centralization of the genetic information*). These fused structures will be referred to as multi-genomes to distinguish them from the fully formed eukaryotic nucleus. They are transitionary in form between somewhat autonomous prokaryotic hybrid cells produced by horizontal gene transfer and a nuclear structure that is totally dependent on the rest of the cell for its maintenance (prokaryotic genomes➔ hybrid prokaryotic genomes➔ multi-genomes➔ nuclei, Fig. 4a). The multi-genomes carry genetic contributions from archaeal and bacterial parents (as does the eukaryotic nucleus, Fig. 1), and can be considered as Bacteriarcheons.

Cell fusion is a widespread phenomenon. The fusion of eukaryotic cells to form syncytia is well known. L-form cells reportedly undergo spontaneous fusion [138]. Similarly, the fusion of bacterial protoplasts is well documented, although agents to promote fusion, such as polyethylene glycol, are needed to promote the process [139, 140]. The fusion of two strains of *Bacillus subtilis*, for example, often generated diploid products [141, 142], with varying degrees of stability [139, 141, 142]. Merged biparental protoplasts appear to have arisen spontaneously, but transiently, with the fusion agent required for their progression to more stable fused cells [141]. In laboratory settings stable intergeneric bacterial protoplast fusions can occur (see, for example [143]), and, although there is little or no literature on the matter, it seems possible that interspecies and intergenic L-form cell fusions could also occur.

Loss of the cell wall may have been progressive with, initially, only a subset of cells in the ensemble surviving without walls. Indeed, if loss of the ability to form cell walls also impeded secretion of matrix polysaccharides, mixed populations of walled and wall-less cells may have been necessary to maintain the structure of the matrix. The cell membranes of the fused L-form-like multi-genomes, which enclose the chromosomes of the multi-genome, perform a similar role as the nuclear membrane in fully eukaryotic cells, that is, they separate the genomic material of the ensemble from the proxy cytoplasm. As the fused multi-genome evolves to an increasingly nuclear-like structure, so too its membranes will increasingly evolve properties associated with the nuclear membrane (see *Assembly of a primitive nucleus*) and possibly later further differentiate towards an endoplasmic reticulum-like structure (see *Membrane enveloped organelles*).

It is unlikely that cell fusion would immediately incorporate all L-form cells in the ensemble into a single unified multi-genome structure but would, instead, result in a mixed population of mini-multi-genomes. Prior to their final fixation as primitive nuclei, the L-form multi-genomes would remain as fluid structures, undergoing fusion and fission events, like those still seen in mitochondria, with the population of multi-genomes co-existing and competing for survival (persistence and duplication) in the matrix. Genetic information at the multi-genome stage is carried on several distinct chromosomes within each multi-genome rather than a single chromosome. Thus, the physical merging of cells in the matrix to generate multi-genomes provides a potential point of origin for the evolution of nuclei with multiple non-identical chromosomes (Fig. 4b). Similar processes of cell fusion [144] or the formation of syncytia [145] have been suggested previously to account for the origin of eukaryotic nuclei with multiple non-identical chromosomes.

The insertion of introns into the coding sequences of the emerging nuclear genomes is allowable under assumption 4 (*intron invasion*) and linearization of their DNA content is allowable under assumption 5 (*chromosome linearization*). The transfer of matrix eDNA back to the genome, particularly if reinserted in a non-coding format, has the potential to add “bulk” to the genomic DNA and so provide an early substrate for the evolution of ancestral long intergenic sequences. Genomes constructed in this fashion would be syncretic, that is, they would be composed of a mosaic of genes selected from a range of bacterial and archaeal groups, as is, in fact, seen in extant eukaryote genomes (Fig. 1) [7, 8, 65, 146].

#### Potential consequences of genome merger and multi-genomes for the development of cellular complexity

Lynch [121]. An increase in cellular complexity is required by the third-space model in order, amongst others: (i) to handle the challenges imposed by structural reorganization, for example, the coordination of multiple chromosome replication or the removal of introns during mRNA processing; (ii) to take advantage of new opportunities that arise from structural reorganization such as the development of cytoskeletal mobility or predatory phagocytosis [147].

The structural and genomic complexity of eukaryotes has been rationalized previously as a consequence of the mitochondrial endosymbiosis “powering-up” the developing cell [37, 40, 75, 76], or as a corollary of population genetics in a small effective population coupled to sub-functionalization [120, 121] (which is defined as occurring among paralogous genes “when both members of a pair are partially degraded by mutations to the extent that their joint expression is necessary to fulfill the essential functions of the ancestral locus”). In a population-based third-space model, the sum of the genomes would be highly redundant with multiple copies of genes for the same function obtained from the disparate partners, whether considered as a set of independent genomes embedded in a matrix, or later as multi-genomes (progenitor nuclei). Redundant genes would be available for functional repurposing (by neo-functionalization or sub-functionalization) allowing gene families to form and expand. Successful innovations would spread through the ensemble by competitive selection, and by horizontal gene transfer, with productive mutations becoming the shared property of the ensemble. (Depending on the nature of the innovation, these could be adopted by all cells in the ensemble, or by subsets, for example only archaeal cells or only bacterial cells). Moreover, if two or more advantageous genetic innovations with potential to mutually reinforce, (that is, show positive epistasis), were to arise anywhere within the ensemble there is a greater chance of their eventual transfer into a single genome as the result of horizontal transfer than in a widely dispersed population, thereby further potentiating the acquisition of molecular complexity among the emerging cells. Comparative genomic analyses confirm an extensive elaboration of gene families by paralogous expansion (that is, direct gene duplication) and pseudoparalogous expansion (that is, genetic multiplication by horizontal gene transfer) early in the evolution of eukaryotes [148].

Populations of monoploid hybrid cells and, later, of polyploid multi-genomes, allow for spatial partitioning of risk. (Polyploid here is simply taken to mean more than one chromosome). Thus, in a population of monoploid hybrid cells embedded in a stable third-space matrix, a genetic crisis arising in one member of the ensemble need not compromise the performance of the partner cells while the “damaged” cell could still be suppressed or eliminated by negative selection. The third-space matrix provides, therefore, an error-tolerant microenvironment in which to “test” genetic innovations and escape the negative consequences of deleterious genetic variations [133]. Similarly, the genetic redundancy of the multi-genome would be protective since, when the polyploid multi-genome has multiple redundant copies of a functionally equivalent gene, deleterious mutations among these genes can be compensated for by the “good” copies, but an advantageous mutation could still be selected for. The damaged genes will still be carried but are now available for eventual repurposing or silencing by mutation (creating bulk in the genome) or removal by deletion. The multi-genome must, however, possess ways to cope with unequal segregation (segregation load) and the accumulation of deleterious mutations (mutation load).

#### Evolutionary traps, the negative consequences of horizontal gene transfer and ploidy, the emergence of mitosis

Horizontal gene transfer will transmit deleterious genes as well as potentially harmful mobile elements, (possibly including catalytic type II introns) [149]. As noted above, with ensemble cells in the monoploid state, and prior to the formation of the multi-genome, selection would tend to suppress these harmful mutants or gene combinations. The multi-genome stage, however, represents a form of polyploidy where, even though remaining “good” genes compensate for “bad” genes on other chromosomes, the less advantageous copies remain free to accumulate to the possible detriment of the population.

Polyploidy occurs in some Bacteria [150] and Archaea [151], as well as chloroplasts [152]. Its potential roles in the evolution of the early nucleus and mitosis have been investigated by computer simulation [153]. Briefly, polyploidy, by itself, provides a short-term advantage over monoploidy, but represents an “evolutionary trap” as polyploids prove less successful than monoploids in longer-term simulations [153]. Homologous recombination, either by gene conversion (asymmetrical recombination) or crossing-over (symmetrical recombination) improves the predicted fitness of polyploid cells [153]. Gene conversion is known to maintain the genetic fitness of polyploid chloroplast genomes [152]. Ploidy cycles, with genomes transitioning from a polyploid to a monoploid condition and back again, improved long-term fitness by allowing for purging of effete genomes during the monoploid homozygous state [153]. Mitochondria provide a potential model for ploidy cycling [154] since fused mitochondria are polyploid, whereas fragmented mitochondria, following fission, have low ploidy or are monoploid. Comparable ploidy cycles of fusion (polyploid) and fission (monoploid) are likely among early multi-genome structures since the mitochondrial fusion/fission lifecycle was taken as a model for the formation of multi-genomes (“*allowable assumption 2*” above). High frequency horizontal gene transfer, between closely related cells, which would be favoured in third-space models, also improves long-term fitness of polyploid populations [153]. By far the greatest improvement in long-term survival of the polyploid simulations came with accurate chromosome segregation between daughter cells, that is, with the innovation of a form of primitive mitosis. This eliminated the segregation load and was most effective when also combined with chromosome exchange, gene conversion and crossing-over [153]. Extrapolating from the simulation studies of Markov and Kaznacheev [153], similar evolutionary considerations would apply during the transition of polyploid multi-genomes from amitotic bags of chromosomes to primordial mitotic multi-chromosomal cell nuclei. Monoploid prokaryotic cells accurately segregate duplicated chromosomes [155] as well as low-copy number plasmids [156], and, presumably, similar mechanisms seeded the evolution of more elaborate machinery for segregation in multi-chromosomal multi-genomes and, ultimately, the nucleus.

#### Assembly of a primitive nucleus

Assembly of the multi-genomes into a singular stable nucleus would help overcome problems that would be associated with coordinating the division of the multi-genomes with the division of the emerging eukaryotic cell. Moreover, retention of a diploid state maintains the protection against recessive mutations afforded by polyploidy.

Mitosis is highly variable [157]. It may be closed, with the nuclear membrane retained throughout, or open, with the nuclear membrane breaking down and reforming as cell division progresses, with many intermediate forms also described, such as semi-open mitosis [157–159]. If nuclear division originated from fission of L-form-like multi-genomes, their cell-like double membranes would, most likely, remain throughout their division and the progenitor nuclei would therefore undergo closed-form mitosis. This maintains a topological distinction between nucleus and cytosol during mitosis. When mitosis is open (that is, when the nuclear membrane breaks down during mitosis) the tripartite arrangement of the cell can be considered to have transiently devolved to a bipartite state. The nucleolus would develop from the centralization, consolidation and expansion of ribosome assembly processes that already existed in multi-genomes.

#### Fragmenting operons

Prokaryotic genes are typically expressed as part of an operon or gene cluster. Advantageous modifications arising in the multi-genome would, therefore, often be obligatorily co-expressed with other genes on the same operon, some of which might be sub-optimal or engender conflict among related but variant gene products from another chromosome. Moreover, when new molecular pathways incorporate gene products encoded on different chromosomes, mechanisms are required to coordinate their expression. A solution to such problems could be obtained by fragmenting the operons through the interjection of DNA spacer sequences between genes and the development of independent transcriptional regulation. This favours the evolution of long intergenic sequences, eukaryote-like monocistronic mRNA, and complex control of gene transcription.

### Steps towards a eukaryotic cytosol and mitochondria

#### Cell size

If, as proposed, eukaryotic cells developed from populations of prokaryotic cells embedded in a matrix (Fig. 3), the greater size of an ensemble versus single prokaryotic cells would lead ultimately to the greater size, on average, of eukaryotes compared to prokaryotes.

#### Membrane encapsulation and cytosolic takeover

A key step in the transition of the matrix to a cytosolic space is the acquisition of an outer membrane. Biofilm-like matrices are often richly endowed with membranous lipids (in the form of vesicles). When examined by electron microscope tomography, small films from *Myxococcus xanthus* grown in cellulose tubes, for example, are packed with extracellular membranous vesicles and also exhibit a sharply discontinuous 5 nm wide sheath-like outer boundary at the air-water interface that is strongly osmium-staining, consistent with a lipid membrane outer boundary [160]. Although Myxocosccus, being motile and predatory may not be representative, the presence of the outer sheath of the *Myxococcus xanthus* films suggests that, in at least some conditions, microbial ensembles can be enclosed within a membrane-like structure. Extracellular lipids are, therefore, available in prokaryotic communities, and there is evidence that they may form boundary layers under some circumstances.

There are many ways a membrane might form. In the *Myxococcus* example, the membrane-like boundary layer assembles at air-water interfaces and “given the number of secreted vesicles …. and the fact that lipids naturally accumulate at an air-water interface” [160], its formation is thought to be spontaneous. If the size of the colony is relatively small and we allow for growth at an air-water interface, a similar mechanism may be proposed for the formation of the first lipid outer boundary membranes of the emerging eukaryote. In an environment where boundary membranes are unlikely to form spontaneously, an alternative mechanism might involve a subset of cells within the population extending sheet like projections to encase the developing matrix/incipient cytoplasm. Such co-option of the existing membrane construction pathways of the resident cells/multigenomes to build a communal membrane around the matrix ensemble, again, would probably work best in smaller rather than larger matrix ensembles. Alternatively, the need for enclosure might promote the de novo development of a dedicated system for delivering lipids to a bounding-membrane. As the ensemble evolves closer to a cell-like way of life, more refined cellular mechanisms would develop to more reliably and consistently build the enclosing membrane.

Biofilm matrices contain polysaccharide polymers that are absent from eukaryotic cytoplasm. A mechanism of cytosolic-takeover is, therefore, required to remove these polymers and replace them with a cytosol-like structure. Populations of cells within a stable third-space would be expected to exhibit social interactions (see above). Over time, stable coexistence would, in some instances, tend towards strong dependency relationships with some cells adopting increasingly specialized functions, as occurs to some degree in biofilm communities [99]. It is well established that cytosolic bacterial proteins are released extracellularly [85–87], including metabolic proteins such as glyceraldehyde 3-phosphate dehydrogenase, fructose-bisphosphate aldolase, and enolase [161]. As the ensemble of cells embedded in the third-space become increasingly interdependent, a rationale can be envisaged for the development and coordination of increasingly complex shared extracellular biochemical networks composed of secreted proteins. Taken to a limit, extracellular sharing of protein networks would allow for the incremental replacement of the biofilm-like matrix with a nascent protein-based cytoplasm (cytosolic-takeover) over time.

The secretion of specific proteins by prokaryotes [162], occurs by both lytic [163] and non-lytic [164] mechanisms. Co-option of these pathways allows secretion of proteins into the evolving matrix/nascent cytosol. Regulated lysis of bacteria [165] has important roles in the formation of biofilms [165–167]. Released proteins would be likely to accumulate as a concentration gradient from their source, creating a halo-effect of greater cytosolic protein density around their point of origin. Moreover, a bounding membrane surrounding the ensemble (see above) would further prevent the unrestrained dissipation of the released proteins. Initially a mixed matrix would arise, partly of polysaccharide polymers and partly cytosolic. Prokaryotic forms of the cytoskeletal proteins tubulin and actin, and other cytoskeletal proteins, are known [168, 169]. Their incorporation into the transitional matrices provides building blocks for the eukaryotic cytoskeleton. Progressive depletion of matrix polysaccharide polymers could be achieved by mutational loss of their biosynthetic pathways. Once underway, stepwise selection of genetic variations that favour the function and organization of the transitional matrices would lead to an increasingly eukaryote-like cytosol.

To summarize, once contained within a membrane, the lysis of a subset of cells in the population together with regulated secretion from living cells embedded in the matrix would deposit cytoplasmic material into the matrix. Given time, and with a population linked by obligatory symbiosis (see sections “*Partitioning of protein synthesis to the cytoplasm*” and “*Steps towards a eukaryotic cytosol and mitochondria: shared metabolism*”), these processes nucleate the incremental replacement of the matrix by the cytoplasm. As the ensemble evolves to an increasingly cell-like state more orderly and better regulated mechanisms are acquired to stock the third-space with cytosolic material such that forms ultimately emerge where the matrix fully and permenantly replaced by cytosol.

#### Partitioning of protein synthesis to the cytoplasm

Cytosolic-takeover, with shared protein functional networks in the developing cytosol, in turn, provides a parallel rationale for the incremental redistribution of ribosomal protein synthesis from individual cells to the third-space in order to support these networks (Fig. 4b). Placing the translation of extracellular proteins in the matrix/nascent cytosol is logistically advantageous since, rather than trapping the translation products inside the cells or the multi-genomes of the ensemble, many copies of a matrix protein can be made directly at its extracellular site of action from a single mRNA strand. Moreover, once efficient mechanisms for protein import arise, one copy of matrix mRNA and associated matrix ribosomes can simultaneously provision many cells or multi-genomes within the ensemble. The cells of the ensemble would initially retain their own ribosomes, while also contributing to the contingent of matrix ribosomes. As the ensemble evolves towards a more unified cell-like lifestyle, protein synthesis would increasingly devolve to the matrix/nascent cytosol and away from the ensemble cells/multi-genomes (Fig. 4b). This is analogous to contemporary mitochondria where, even though mitochondria are equipped with their own ribosomes, most mitochondrial proteins are translated by cytosolic ribosomes from genes farmed out to the nucleus. Multi-genomes, by contrast, devolved mRNA translation to the cytosol, as per the mitochondria, but retained their genetic complement.

Cell lysis, vesicle budding, or leakage across an L-form plasma membrane might, in the first instance, seed the matrix with ribosomes and mRNA. Regulated lysis of bacteria [165] contributes to the formation of biofilms [165–167], and it has been suggested that the coordinated programmed cell death of a subset of cells in a biofilm population is an “altruistic” event in the biofilm lifestyle [170], a concept that may be applicable to third space models. The closest contemporary equivalent for the lytic extracellular release of a large structure, such as ribosome, is probably the dispersal of bacteriophage from their host cell. Selection to make the mechanism increasingly efficient would lead incrementally to a more eukaryotic-like system of ribosomal manufacture and transport. Bacteria secrete RNA, although in *E. coli* the RNA is mostly in the form of small degradation fragments representative of a cross section of intracellular RNA with both vesicle-associated and vesicle-free extracellular RNA being detected [171, 172]. There is at least one example of extracellular RNA playing a role in biofilm formation [173]. The redeployment of protein synthesis into the third space is a step in the transition towards an increasingly cytoplasmic-like function.

It is likely that cross-system incompatibility would ultimately favour the selection either of bacterial or archaeal matrix ribosomes rather than a hybrid of the two, (this is effectively a manifestation of the “complexity hypothesis” [174, 175]). The eukaryotic ribosome is archaeal in origin [14]. If it is assumed that the greater complexity of the eukaryotic ribosome provides advantages over either the archaeal or bacterial structures, then co-opting the already highly elaborated archaeal ribosome would be a more parsimonious route to achieve the requisite complexity of the eukaryotic ribosome than through a bacterial path. The archaeal nature of the ribosome requires transfer of at least one archaeal chromosome, or a large part of it, into the eukaryotic nucleus, which would, in addition, carry with it the genes required for other archaeal-type information processes of the eukaryotic cell. Once the emerging eukaryotic cell adopted archaeal-like ribosomes, other systems closely linked to the ribosomes would be preferentially selected from archaeal precursors in order, once again, to minimize functional conflict between complex molecular mechanisms that might occur in hybrid structures. Consistent with this concept the nucleolar core structure is archaeal [176], including the small nucleolar RNAs (snoRNAs), although the nucleolus has also acquired significant bacterial content.

#### Steps towards a eukaryotic cytosol and mitochondria: shared metabolism

It has been proposed, based on calculations of “energy per gene”, that eukaryogenesis could occur only after the evolution of specialized energy generating organelles, namely the mitochondria [37, 38]. This is taken to support the prokaryotic endosymbiosis hypothesis. Others have argued against this conclusion [177, 178]. Both aerobic [24–26] and anaerobic [78] origins for the mitochondrial progenitor have been advanced. Aerobic mitochondrial progenitors may have served first to scavenge oxygen and protect anaerobic partner cells from oxygen toxicity, a proposal known as the ‘ox-tox’ hypothesis, [25], with ATP-production as a secondary outcome. Biofilms exhibit localized aerobic and anaerobic regions [100] such that, in a third-space model, an initial role for mitochondrial progenitors in maintaining sub-toxic matrix oxygen levels (ox-tox) conforms with the biological properties of a prokaryotic ensemble. Initially, in a third-space model, each member of the ensemble generates its own energy needs. Bacteria in liquid culture reportedly release ATP into the medium [179, 180], although they lack dedicated ATP translocases [25]. Differential levels of ATP secretion would allow a subset of cells in the ensemble (presumably with an alpha-proteobacteria genomic bias) to evolve towards generating excess (shared) energy (ATP), possibly in return for some other benefit, (such as nutrient support), obtained from the matrix community. These cells would progressively assume a mitochondrial-like ATP-generating role, eventually devolving most non-ATP generating functions to the matrix and multi-genomes, while acquiring dedicated ATP/ADP exchange [25] and protein import systems [181]. Devolution of non-ATP generating functions from the emerging mitochondria to the rest of the ensemble further reinforces the logistical advantage of a matrix-based ribosome protein biosynthesis system to support the emerging mitochondria, while extracellular ATP from nascent mitochondria provides a rich energy supply for the matrix/cytosol protein networks and other cells/multi-genomes in the ensemble. Moreover, as the mitochondrial precursors became increasingly dependent on other cells in the ensemble for most functions other than generating ATP they would, over time, shed unused genes and reduce the size of their own genome. Since the cells providing proteins to the mitochondrial precursors do not have to be alpha-protebacteria, this may underlie the surprising mosaicism of the mitochondrial proteome. Efficient ATP generation requires a large membrane surface area across which to perform chemiosmosis. By remaining as semi-autonomous membrane-defined structures within the emerging cell, the nascent mitochondria retain membrane surface area with which to mount highly productive ATP production. Neither endosymbiosis nor phagocytosis is required, with the nucleus and mitochondria evolving side-by-side from a population of cells embedded in the matrix (Fig. 4a).

The bacterial dominance of essential metabolic and operational processes in the eukaryotic genome can be understood in a third-space model by assuming that the transitionary matrix state was mesophilic, as are most extant bacteria and eukaryotes. Archaeal metabolism is often adapted to extreme growth conditions, and those archaeal lineages that survive well under mesophilic conditions appear to have acquired this ability through the evolutionary co-option of mesophilic bacterial metabolic pathways following large-scale horizontal gene transfer of bacterial genes [69]. Some metabolic pathways in eukaryotes are, nevertheless, archaeal in origin. For example, eukaryotes and archaeal cells use the mevalonate pathway to synthesize isoprenyl lipids, (but generate different lipid families from the isoprenyl subunits), whereas bacteria use the unrelated methylerythritol 4-phosphate pathway [182]. Thus, in a third-space model, genes would be selected not because of their archaeal or bacterial origin, but because they were “fit for purpose”.

#### Genome merger and the loss of archaeal membrane lipids

Merger of archaebacterial genomes and bacterial genomes allows for the retention of archaebacterial genes while abandoning archaeal membrane lipid chemistry. Archaeal lipid membranes are thought to be cytoprotective in extreme environments. As most eukaryotes have a mesophilic lifestyle, the selection of bacterial over archaeal-type membrane lipids by the emerging eukaryote might, therefore, reflect the preferential selection of bacterial cell membrane lipids as they are more suitable for a mesophilic existence. Alternatively, it might result from a simple numerical predominance of Bacteria over Archaea in the early matrix ensemble.

#### Membrane-enveloped organelles

Eukaryotic cytoplasm contains a labyrinth of membranous sub-cellular tertiary structures (perinuclear space, the endoplasm reticulum, Golgi lumen and the lysosome). These spaces are in contact with the extracellular environment through fusion events with the plasma membrane and play a central role in the import/export economy of the cell. Topologically they form an interface between the inside of the cell and the outside. In the topological model proposed here, these are tertiary spaces, that is, spaces that are derived from the secondary spaces (see section *Minimal cellular topologies*). They could result from in-folding of the plasma membrane or out-folding of a primitive nuclear membrane, either of which are compatible with a third-space hypothesis. Prokaryotes in biofilms shed lipid encapsulated membrane vesicles [88, 160, 183, 184], a process that, in archaea and eukaryotes, is mediated by ESCRT-II-(endosomal sorting complexes required for transport)-homolog proteins [185]. In a third-space model, membrane folding or shed vesicles from the multi-genomes or primitive nucleus, perhaps co-opting ESCRT-machinery proteins, may have provided structural substrates for the evolution of early-membrane enclosed cytosolic structures, such as the endoplasmic reticulum. *Lokiarchaeota* possess a rich complement of RAS-family small GTPases [18], some of which may have provided the evolutionary substrate to develop regulatory proteins of eukaryotic vesicular transport and membrane folding. In this model, the endoplasmic reticulum would have developed from the primordial nuclear membrane, rather than vice versa. This is consistent with the physical continuity between the double-walled nuclear membrane and the endoplasmic reticulum, together with the structural similarities between certain proteins of the nuclear pore and proteins involved in cytoplasmic vesicle transport [186]. Interestingly, there are similarities between export of unfolded proteins into the bacterial periplasm and the eukaryotic transport of unfolded proteins into the lumen of the endoplasmic reticulum [162], which is compatible with a periplasm-like origin for protein import into the endoplasmic reticulum.

#### Cellular third spaces

The model developed above employs a non-cellular third space. Other third spaces may be considered, including cellular third spaces. For example, a divergence of bacterial cells into nuclear and mitochondrial spaces, similar to that proposed above, could occur in the prokaryotic endosymbiosis model if the passenger endosymbiont forms a small population within the host. A subset of passenger cells differentiate into L-form-like mitochondrial progenitors and another subset into nuclear precursors, with both contained within the host archaeal space which forms the proxy cytosol. This is, effectively, a three-space prokaryotic endosymbiosis with an animate third space, A strength of this variant of the model is that cytosolic ribosomes will be archaeal by default. Fusion of multiple L-form passenger cells would give rise to transitory polyploid multi-genome-like structures, as above, which would in turn would favour the development of primitive mitosis [153]. It does not, however, overcome the problem of archaeal/bacterial endosymbiosis, or, without further modifications, result in a highly syncretic eukaryotic genome.

### Limitations and advantages of the strong third-space model

#### Limitations

1. Biofilms propagate by dissipation through a unicellular planktonic stage [85–87] (Fig. 3). During the unicellular phase innovations made in the communal matrix phase may be lost by dilution. The emergence of cell-like autonomy in the third-space matrix therefore requires a mechanism to stabilize the matrix in the long term and either allow it to propagate without dissipation or to reassemble with high efficiency and fidelity after dissipation. The third space model is a multi-stage process and, therefore, the degree of stability required need not be the same at each phase. Initially, when the matrix is identical or very similar to a biofilm, stability might be afforded by the reproducible reassembly of the relevant populations of cells after dispersal. This would be based on mutualistic interactions among cells within the matrix. In intermediate stages the presence of a lipid sheath around the matrix would stabilize the resident cell population within a contained space. As the mutualistic dependency of the ensemble becomes stronger, populations that are the most physically stable, or reassemble most efficiently, will have an advantage over less stable ensembles, thereby favouring selection for increasing stability until structural persistence is achieved.
  Physical cell-cell association within the matrix provides a further route to enhance the stability of the matrix ensemble, particularly if these associations significantly enhance the survival of the ensemble members. Short term experiments that employed a biofilm community of two species, (an *Acetinobacter* and a *Pseudomonas*), one of which metabolized the sole available carbon source to a form that the other species could use, found that mutations occur within less than ten days that increase the stability of the communal biofilm (with respect to dispersal after oxygen depletion), promote physical association of the two species and increase overall productivity compared to the same two species grown in a chemostat [187, 188]. Biofilms can undergo multicellular fragmentation allowing propagation without a planktonic stage [189]. Indeed, some multicellular magnetotactic bacteria appear to have abandoned entirely the need for unicellular propagation [190]. Microbial mats provide an example of a long-lasting microbial community.

2. The model does not fully illuminate why the function of eukaryotic genes with an archaeal ancestry are weighted towards information processing and translation. It assumes that systems were selected by competition, the most successful “winning out”, and that the existence of merged genomes (the bacteriarchael multi-genomes) would expose both archaeal and bacterial systems to competition. In some instances, such as ribosome structure, additional hypotheses such as parsimony in achieving complex structures can be invoked to rationalize this bias. Once any important part of the information processing mechanism in the emerging cells had been established from archaeal modules, other linked modules would be selected preferentially from archaeal sources in order to minimize cross-system incompatibility, as is seen in the archaeal “core” of the eukaryotic nucleolus [176].
3. Eukaryotic cells are monophyletic [8], and this is taken as evidence of a singular, or very rare, prokaryotic endosymbiotic event at the origin of eukaryotes. At face value, the third-space model favours multiple (polyphyletic) origins for eukaryotes. It is, however, easy to construct mechanisms that would facilitate monophyly in third-space models. For example, successive steps towards a fully eukaryotic cell might have to cross progressively ascending thresholds of improbability. Similarly, the candidate eukaryotic progenitors could be winnowed to a very small number if only one step is rare, for example cellular breakout, or, later, the evolution of highly specialized but important eukaryotic behaviors such as mitosis and meiosis.

#### Advantages

1. The model avoids vanishingly rare events, such as archaeal/bacterial endosymbiosis. Similarly, complex eukaryote-like behaviors such as phagocytosis are not required.
2. By facilitating horizontal gene transfer in the matrix, the resulting merged genome is syncretic, comprising genes drawn from a cross section of species. This is consistent with the complex source of genes of prokaryotic origin found in the eukaryotic genome.
3. The genetic redundancy within the matrix ensemble and, later, in multi-genomes, facilitates the development of extended gene families and the concomitant emergence of cellular complexity.
4. The model is compatible with eukaryote-like genome reorganization including linear chromosomes, frequent introns and extensive inter-genic sequences.
5. Cells with multiple non-identical chromosomes emerge as a natural property of the model with mitosis arising as a corollary of polyploidy.
6. Nuclear structures arise in a step-wise fashion from the membranes and internal structure of the multi-genomes.
7. The model mitigates the genetic crises likely to accompany genomic reorganization by providing an error-tolerant environment to buffer disruptive innovations while retaining constructive innovations.
8. The model provides a potential point of origin for the initial partitioning of ribosomes out of the cells of the ensemble or multi-genomes and into the matrix/cytoplasm.
9. The model enables emergence of mitochondria and nuclei by division of labour among the ensemble of matrix embedded cells.
10. It provides a mechanism for significant archaeal contribution to the eukaryotic genome, while relinquishing archaeal membrane structure.
11. Membrane vesicles or folding may provide substrates for the development of complex sub-cellular membrane structures.
12. Although not explicitly considered here, the model should be sufficiently adaptable to incorporate other spaces including viruses [81] or “ghost” lineages such as the hypothesized chronocyte [33]. It should also be possible to modify the model to formally include dependencies (such as syntrophy, parasitism, predation) as part of the initial conditions.

#### Prior examples of third-space models

Although third-space models of cellular evolution are not widely studied, prior examples exist in the literature. These differ from the model developed here as each space consists of a single species rather than a mixed population, and the association of spaces may occur sequentially rather than in parallel. Examples include:

1. In the syntrophic model of sequential endosymbiosis [79], the first space is provided by bacterial cells. These acquire a second archaeal space that establishes the nucleus. The merged organism then acquires a third distinct bacterial space that establishes the mitochondria.
2. In the chronocyte model of eukaryogenesis [33], a hypothetical lineage, the chronocyte, which is neither bacterial nor archaeal, forms one space. Uptake of the archaeal progenitor of the nucleus provides the second space, and uptake of the bacterial progenitors of mitochondria forms a third-space.
3. In the only well-defined example of bacterial-bacterial endosymbiosis, a small population of gamma-proteobacteria resides inside a beta-proteobacterium inside specialized eukaryotic insect cells of a mealybug [61]. This can be thought of as a three-space system, representing a set of nested containing spaces where the insect primary space contains the beta primary space which contains the gamma primary space. (The arrangement can, of course, equally be interpreted as a five-space model, considering the insect cell as three spaces; nucleus, mitochondria, and cytosol). Thus, while prokaryotic endosymbiosis in free-living organisms remains elusive, it can be maintained under very special circumstances inside a sufficiently supportive and persistent third space. This organization differs from the eukaryogenic third- space models of Fig. 2 as it breaks criterion 5 of the “*minimal cellular topologies*” but, nevertheless, provides a real world example of how genes and cellular functions can be redistributed among partners in a simple population-based third-space system [61–63].

## Discussion and conclusion

The third-space hypothesis is based on two arguments. Classes of potential eukaryotic origin were identified by analyzing the underlying structural logic or topology of eukaryogenesis assuming a derivation from simpler cells. Two classes of solutions are compatible with the established models of autogenic or prokaryotic endosymbiotic eukaryogenesis (Fig. 2a, b). A further class of solutions, (Fig. 2c), the third-space models, provides an additional framework for the eukaryote transition that has not been extensively investigated. It differs from other hypothesis for eukaryotic evolution as it treats the environment in which the evolutionary transitions take place (the third-space) as operational, contributing fundamentally to the transitionary process, rather than as a passive carrier or bystander. The second part of the argument investigates the kinds of process by which a eukaryotic cell-like structure might emerge from the third-space model (Figs. 3 and 4). Hallmarks of eukaryotic cells were derived from the model after incorporating a limited set of “allowable” assumptions that are based on properties of cells alive today. A biofilm-like third-space was proposed, although the logic of the third-space model is applicable to other potential third-space environments. The strong third-space solution does not necessarily require either endosymbiosis or phagocytosis. Exosymbiosis takes the place of prokaryotic endosymbiosis (which is conspicuously rare), or other form of cellular engulfment. Horizontal gene transfer, and the consequent merging of genomes (Fig. 1), is integral to the model which predicts that the genetic makeup of the earliest eukaryotes was syncretic, highly redundant and reflected the antecedent ecological makeup of the matrices in which they formed, with dominant species in the ensemble contributing most to the merged genome. The ensemble of matrix and cells that comprise the third-space model must be sufficiently stable for modifications to persist and propagate (Figs. 3 and 4). Over time the cells that live within the stabilized matrix lose their individual identity while the ensemble itself, or a sub-group within the ensemble, slowly acquires a new identity as the precursor of eukaryote-like cells (Fig. 3). Several properties of eukaryotic cells, such as increased cell size, nuclei with multiple chromosomes, mitochondria, cytosolic ribosomes and a bacterial-like membrane lipid composition can be derived as rather straight forward predictions from the model. Genomic redundancy in the ensemble foreshadows the onset of complexity in the final eukaryotic cells. Putative routes towards primitive mitosis can be postulated by combining the third-space hypothesis with a polyploid model for chromosomal origin [153] which further predicts that some of the characteristic properties of the eukaryotic nucleus, such as mitosis and chromosome linearization, emerged initially as escape routes from an evolutionary trap imposed by polyploidy. Introducing a transitionary multi-genome stage between prokaryotic cells and the eukaryotic nucleus allows parallels to be made between nuclear and mitochondrial development, as both the multi-genomes and mitochondria resemble L-form cells, and both undergo phases of fusion and fission. Mitochondria retained this state, presumably to preserve the extensive membrane surface area needed for efficient chemiosmosis, while multi-genomes progressed towards a unified nuclear organelle to avoid conflicts that would arise between cytokinesis and the uncoordinated division of a population of multi-genomes. The weak and intermediate variants of the model are more closely related to conventional models of eukaryogenesis, but emphasize communal interactions mediated by the third-space.

**Fig. 5:**
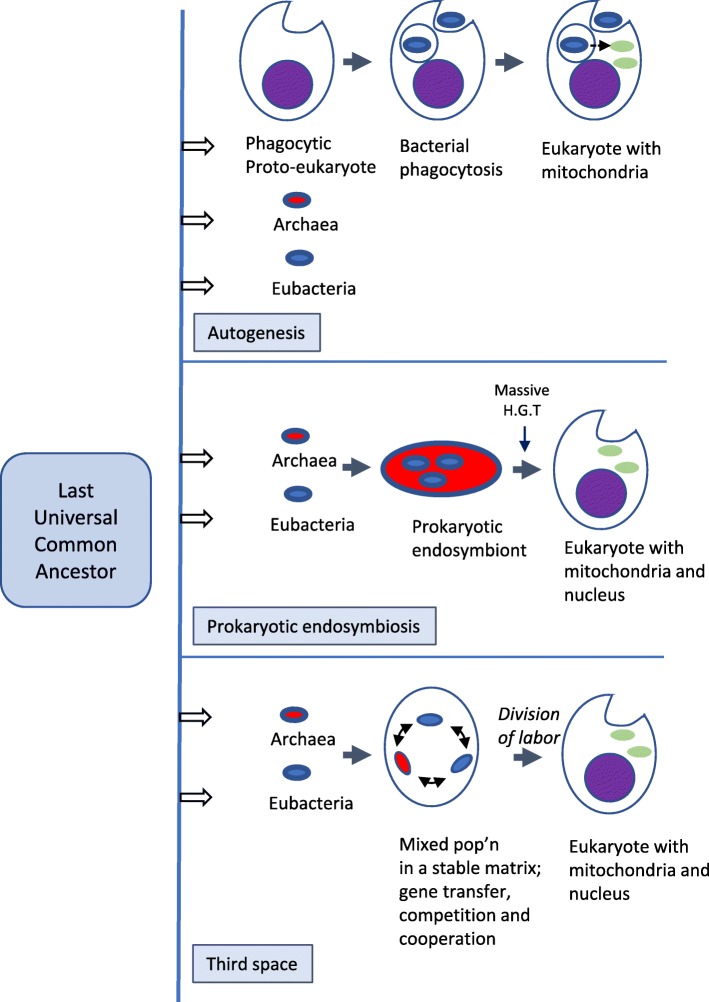
A comparison of autogenous and prokaryotic endosymbiotic models of eukaryogenesis with third-space model. In the autogenesis model, Proto-eukaryotes, Archaea and Bacteria all emerge from a last universal common ancestor. The Proto-eukaryotes then acquire a bacterial mitochondrial precursor by phagocytosis. In prokaryotic endosymbiosis, only Archaea and Bacteria connect directly to a last universal ancestor. Eukaryotes emerge as the result of endosymbiosis of an archaeon (the host) with a eubacterium (the passenger) that subsequently evolves into the eukaryotic mitochondrion. Massive horizontal gene transfer (H.G.T) then transfers the majority of prokaryote-derived genes to the emerging eukaryote. In the strong third-space model only Archaea and Bacteria link directly to a last universal ancestor. By co-existing in a closed and stable third-space, mitochondria and nuclei emerge by division of labour, gene transfer and cell fusion

Models of eukaryogenesis are compared in Fig. 5. Autogenic eukaryogenesis is “gradualist” [191], that is, it occurs by progressive accumulation of change, whereas prokaryotic endosymbiosis hinges on a discrete event, the singular capture of the mitochondrial progenitor, and is, by definition, founded on co-dependence, or cooperation, between the endosymbiotic partners [191]. Co-dependency plays less of a role in autogenic models. The strong third-space model differs from either of these paradigms as it is gradualist and postulates critical roles for a balance both of cooperative and competitive relationships among the participating elements. The version of the third-space model elaborated here circumvents some of the problems of the autogenic and prokaryotic endosymbiotic models of eukaryogenesis, (see *“Advantages of the third-space model”*) but raises its own questions (see “*Limitations of the third-space model”*).

Future research to improve or refute the third-space hypothesis might include modeling studies to set boundaries on some of the proposed processes. Aspects of the model might be reproduced in the laboratory by experiments to investigate, for example, long-term bacterial and archaeal gene flow in stabilized matrices, the long-term behavior of L-form cells in stabilized matrices and the genetic and functional consequences of forcing differing L-form cells to merge in a stabilized matrix. Ultimately, it may be possible to recreate the transitionary matrix state in the laboratory and, by doing so, design simple novel synthetic life forms. Third-space models provide an internally consistent framework for a family of largely unexplored solutions to the problem of eukaryogenesis (Fig. 2c), some of which (Figs. 3 and 4) obviate the problematic need either for prokaryotic endosymbiosis or phagocytosis as primary mechanisms for eukaryotic evolution. Analyzes of early eukaryotic evolution should, therefore, include tests against the third-space hypothesis.

## Reviewers’ responses

I would very much like to thank the reviewers for their thoughtful and very helpful comments and criticisms. Their consideration, patience and time is greatly appreciated.

Since submitting the original draft, a paper has been published outlining the first culture of an Asgaardian organism. This has been added to the introduction during revision (Starting “To date, only one Asgardian species has been cultured,……”

### Reviewer’s report 1: Damien Devos, Affiliation European Molecular Biology Laboratory (EMBL), Heidelberg)

***Reviewer Summary***

*In the manuscript “Division of labour in a matrix, rather than phagocytosis or endosymbiosis, as a route for the origin of eukaryotic cells”, the author proposes a novel model for the origin of the eukaryotic cell. The author discusses the introduction of a novel third-space that could be have played an import part in the development of the ancestral eukaryotic cell. In summary, the author proposes that biofilms have played a structural role in the development of the eukaryotes. The issue is of course of interest, of actuality and still an important unresolved question. The manuscript is well written, the ideas developed are of interest and well argued. I believe that the manuscript is of interest to a wide community of readers.*

**Reviewer recommendations to authors.**

*Main issues: One of the disturbing point is that the manuscript keep calling archae and bacteria as “Archaea” and “Eubacteria”. This is an historical mistake, as the term eubacteria was introduced to differentiate them from archaeabacteria (by Woese). But now, these terms have been defined as “Archaea” and “Bacteria”. Still mixing both is a conceptual mistake. This has to be fixed thourough the manuscript.*

Author response: This was careless and has been changed throughout.

*The model introduce the concept of “third-space”, a novel class of frameworks for the origins of complex cellular structures. In particular, it has three requirements L264. The third one is to be “more than a passive arena” for evolution. Please develop as this is quite limited. How so? How could it promote evolution?*

Author response: *New text has been added to hopefully clarify this point. Section “What are the three spaces?” Paragraph two starting “As the third space evolves from a matrix ….” to “tie together the cellular elements of the ensemble as a coordinated system.”*

*However, my main issue is with the point ii, L265, where the third-space is required to be ‘stable’ enough to provide the opportunity for eukaryotes to emerge. How long exactly would that be? How long do the author estimate that the presence and stability of the third-space would be required? And related to this, how long are biofilms known to be stable? Would it be realistic to think that particular biofilms could be stable for so long? If so, in which environment? Please develop this fundamental issue. Examples from biofilms or microbial mats would nicely illustrate the point.*

Author response: *This is difficult and I think that the reviewer has raised a very important question. I have added new text to discuss the question of stability in section “Limitations”, first paragraph starting from “The third space model is a multi-stage process and the degree of stability required need not be the same at each phase…” to paragraph 2 ending “Microbial mats provide an example of a long-lasting microbial community”. Stability, in this context, can be broken into two types, namely physical stability of the matrix ensemble, and/or its ability to efficiently reassemble if it is dispersed. Furthermore, the hypothesis is clearly for a progressive multi-stage transition, and therefore the degree of stability can ramp-up as the process continues. Initially it would be sufficient for the ensemble to efficiently reassemble, presumably driven by obligate mutualistic interactions. As the accuracy of reassembly becomes increasingly a dominant aspect of the system a shift towards physical persistence of the ensemble becomes favourable, possibly aided by the formation of an outer membrane.*

*Please also detail how this proposal differ from refs* 95 and 96.

Author response: *New text has been added in the section “Extracellular matrices as models for the third-space”, paragraph two" Starting “Jeckely (101) proposed that early eukaryotic evolution occured in a social biofilm-like context…” until “…. compaction or streamlining of structures to generate an entity more closely resembling a eukaryote”*

*Also, if the third-space is required to be so stable, why can we not find any intermediary forms in current biofilms? This deserves mention.*

Author response: *I suspect that the lack of intermediate forms is simply because they were replaced by “fitter” increasingly more eukaryotic-like cells as the process moved forward. Also, I am not sure what we would look for to find any residual intermediate forms. As the manuscript is already rather long, and this is not an easy topic to answer succinctly I would prefer not to add it to the text.*

*Most of the model is based on gene exchange inside the “third-space”, assimilating it to a genomic reservoir to support gene exchange L250. However, the chapter (L245–254) is quite theoretical and ill supported by examples. Could such examples be provided by example based on known interchanges in communities and biofilms? Specifically discuss bacteria/archaea interchanges.*

Author response: *I have added new text to expand the discussion of HGT from Bacteria to Archaea in the section "Genomic reorganization towards a eukaryotic pattern, first paragraph “The horizontal transfer of genes between prokaryotes is a fundamental property of their evolution, and has allowed for very extensive genetic mosaicism to occur in some cases, including in inter-domain mosaicism, where both archaeal and bacterial genes are prominent in the same organism (69). For example, the evolution of archaeal Halobacteria was marked by the acquisition of approximately1000 bacterial genes families (67). Interdomain mosaicism is similarly evident among Lokiarchaea species, 33% of their genes are unique to these species, with the remaining 36% of archaeal origin, 28% bacterial and 2% of eukaryotic origin (125). Bacteria may likewise carry a significant archaeal genetic content, thus in Thermotoga maritima, 24% of genes have been imported from archaeal species. Horizontal gene transfer is promoted by growth in biofilm matrices both in Archaea, as has been demonstrated, for example, in Haloferax volcanii (126), and in Bacteria ((127-129)).”*

*A major issue with the proposal is related to the lipid composition of eukaryotic membranes* versus *archaeal ones. Could the author elaborate around line 120?*

Author response: *I have added new text “If the host cell of the ancestral endosymbiotic partnership was archaeal, as is often proposed (33, 63, 77), then, at some stage of eukaryogenesis, it must relinquish its characteristic archaeal membrane-lipid biosynthetic pathways in favour of those of the bacterial passenger cells. The mechanisms and evolutionary rationale underpinning this transition remain unclear.”*

*In the allowable assumptions, I have a few responses of interest. L303, L-form bacteria are discuted and it is stated that L-form cells have been proposed to have played a significant role in early cellular evolution. However, this is a proposal about early cellular division, not about division of the eukaryotic cell. Please clarify or remove.*

Author response: Yes, that is correct. I have removed the statement.

*L 306, fusion of mitochondria in the eukaryotic cell is assimilated to fusion events that could have occurred between the participating cells within the third-space matrix. There is however a major difference in the fact that participating cells in the third-space matrix would be of different or differentiating types. To the best of my knowledge, fusion between different cell types have not been observed. Please response.*

Author response: *At fusion stage the cells in the matrix ensemble have lost cell walls and adopted an L-form lifestyle. Not much is known about fusion of L-form cells, although one report does mentions that L-form cells fuse in culture. Fusion between protoplasts of cells of different type can be accomplished. However, it should be pointed out that these fusions are in the lab not in the wild. I have added text to discuss these points in section “Genomic reorganization towards a eukaryotic pattern”, paragraph 5, starting “Cell fusion is a widespread phenomenon” to “…. and intergenic L-form cell fusions could also occur.”*

*. Related to this point, L 374 states that “the nascent nuclear membrane arises from the cell membranes of the fused L-form-like multi-genomes”. This is an important jump and worth much more discussion. Why would membrane surround the DNA, and how do the author envision such transition.*

Author response: *The DNA resides within the resident cells of the matrix, even after loss of cell wall and fusion events occur. The formation of nuclear membrane. Some of these resident cells specialize as primitive nuclei, and therefore the nuclear membrane is an adaption of their earlier cell membranes. It is not generated de novo. The discussion of this point has been expanded in section “Assembly of a primitive nucleus”.*

*Relatedly, how could membrane then form around the third-space?*

Author response: *This was not well explained in the original manuscript and was relegated to a few sentences near the end. I have now inserted a new subsection that deals with this question at more length. See first paragraph of section “Membrane encapsulation and cytosolic takeover”.*

*Another issue is with the assumption that eubacteria outnumbered archaea in the matrix to justify the eubacterialization of the archaeal component of the matrix. Why would the author assume this, if not to justify a posteriory a known bias in eukaryotic genomes? If this is so, this is a deduction, not an assumption.*

Author response: *The rationale for the assumption/deduction is now given in greater detail in Section “Genomic reorganization towards a eukaryotic pattern”, paragraph 3, starting “While transfer of genes can occur in either direction….”*

*It would be important to clarify how an increase in cellular complexity is required in order to “overcome problems that occur when molecules brought together from different lineages fail to interlock efficiently”. Please detail and response.*

Author response: I agree that this was not very clear, and it has now been deleted.

*However, the most important point to me, is related to the transformation of the cytoplasm of one prokaryote into the nuclear AND of the extracellular space into the cytoplasm. Both are dramatic modifications that are not explained at all in the current manuscript and that deserves much more details as they are so central to the proposal. L 508 and others and also figure 3 b.*

Author response: *The transformation of the matrix to a cytosol is now discussed in much more detail in the new section “Membrane encapsulation and cytosolic takeover.” In the original manuscript the transition from the matrix to the cytosol was referred to as “matrix replacement” and discussed too briefly near the end of the manuscript. I have changed the name of this process from “matrix replacement” to “cytosol takeover” in order to better emphasize the nature of the transition. In effect there is a slow transfer of cytosolic components from the resident cells into a membrane enclosed matrix resulting in a series of intermediate or transitionary matrices with increasing cytosolic and decreasing matrix properties. Selection for ensembles with improved matrix properties results in the increasing approach towards a cytosol-like structure enclosed in a membrane sheath. Suggestions regarding how this could occur are presented.*

The formation of the nucleus occurs through the fusion of the L-form cells, a subset of which specialize by division of labour into information storage and processing centres. The text that discusses this has been edited and given a sub-heading. *“ Assembly of a primitive nucleus.”*

*In particular, if the archaea is proposed to become the nucleus, as in other proposals, and stated L694, how typically archaeal lipids are modified to become typically eukaryotic and bacterial lipids in the nucleus?*

Author response: This is, I think a misunderstanding. This section discusses other previously published models that have “third-space” characteristics and does not apply to the model presented here. These other models were presented in order to allow the reader to compare them with the current model and make decisions about their relative strengths.

*L586 deserves more details.*

Author response: *I have revised this text from “The selection of eubacterial over archaeal-type membrane lipids might, therefore, reflect an adaption of membranes to a mesophilic lifestyle or, alternatively, result from a simple numerical predominance of eubacteria in the early matrix ensemble”; to now read “As most eukaryotes have a mesophilic lifestyle, the selection of bacterial over archaeal-type membrane lipids by the emerging eukaryote might, therefore, reflect the preferential selection of bacterial cell membrane lipids as they are more suitable for a mesophilic existence. Alternatively, it might result from a simple numerical predominance of Bacteria over Archaea in the early matrix ensemble.” (in section “Genome merger and the loss of archaeal membrane lipids”).*

*An important issue is that some biofilms are compatible with the existence of a bounding lipid membrane L 655. I find this argument extremely weak and not supported at all by the reference provided. Please revise.*

I agree that the presence or absence of a bounding membrane in the biofilm was not the main point of the paper referenced, however the evidence given in that paper for a sheath around the colonies that takes up osmium stain as expected for a lipid membrane is good. I have revised this to be more “equivocal” about the interpretation of this structure as a membrane, and also to point out the presence of numerous extracellular vesicles in the matrix. This text is now incorporated into section. *“Membrane encapsulation and cytosolic takeover”,* in paragraph one, sentence starting “When examined by electron microscope tomography ….” to “…ensembles can be enclosed within a membrane-like structure.”

*Minor issues: L20, the author states that “the question was addressed by enumerating the classes of potential pathways”. I however see only the two classical pathways, autogenous and fusion, followed by the novel model. There is no ‘enumerating’. I would just remove this line.*

Author response: This has been changed to "The question was addressed by considering classes of potential pathways from prokaryotic to eukaryotic cells…. (In the abstract).

*L 45 states that the age of the first eukaryotes is between 2.1 and 1.84 Ga, “making them much younger than Archaea or Eubacteria”. If we would agree that bacteria are ancestral, there is much less consensus on the age of the archaea. Could the author develop on the question of the age of the Archaea?*

Author response: *This is an important question, but as the manuscript is already very long, I preferred to change the sentence to “making them much younger than prokaryotes”, thereby avoiding the issue, rather than discuss the question at length.*

*Please define at first mention, eg L72, what is meant by a proto-eukaryote.*

Author response: *This has been modified, third paragraph of the Introduction, sentence starting “Proto-eukaryotes would be expected to have several hallmark features….”*

*L421, modify ‘consistent’ as this is not consistent, but this is the same thing.*

Author response: This has been done.

*L479 is in favor of chromosome linearization arguing that two circular chromosomes cannot segregate properly after recombination if the number of crossovers is odd. I believe that this is pure speculation and not supported by the two theoretical analyses used as refs.*

Author response: This has been removed as it was not central to the hypothesis.

*L481 states that mitosis is either closed or open. This is untrue as there are plenty of intermediary, as reviewed in Sazer* et al.*, 2014 curr biol.*

Author response: Yes, this is absolutely correct, I changed the text accordingly to read “Mitosis is highly variable (157). It may be closed, with the nuclear membrane retained throughout, or open, with the nuclear membrane breaking down and reforming as cell division progresses, with intermediate forms also described, such as semi-open mitosis (157-159).”

*L499 states that increase in cell size follows obviously. It is unclear to me, why this should be so obvious. Please detail.*

Author response: *This has been revised to read “4.1. “Cell Size”. If, as proposed, eukaryotic cells developed from populations of prokaryotic cells embedded in a matrix (Fig. 3), the greater size of an ensemble versus single prokaryotic cells would lead ultimately to the greater size, on average, of eukaryotes compared to prokaryotes.”*

*L599, reference* 160 *is NOT the correct one to this statement the correct one is Devos* et al.*, Plos biolgoy 2004.*

Author response: Thanks. This has been changed as suggested.

*Advantage listed a #11, L682 is not supportive as matrix membrane vesicles still needs the ability to fuse in the external environment.*

Author response: This is now changed to "Membrane vesicles or folding may provide substrates for the development of complex sub-cellular membrane structures.

*Figure 4, blue stream left, “ribosomes in prokaryotic cells” is stated twice*

Author response: Thanks for noticing this, it has been changed.

### Reviewer’s report 2: Buzz Baum, Affiliation University College London, Cell biology

**Reviewer summary**

*The origins of the eukaryotic cell remain uncertain. Models can certainly help here. Therefore, we read this paper with interest. In this paper, “Division of labour in a matrix, rather than phagocytosis or endosymbiosis, as a route for the origin of eukaryotic cells”, the author proposes a novel mechanism by which the eukaryotic cell may have arisen from a community of prokaryotes living within a shared environment, or biofilm, which the author calls the “3rd space”. The author justifies the need for this new perspective by stating that there are “irreconcilable differences” between existing hypotheses, and on evidence that a large part of the eukaryotic genome cannot be attributed to the two proposed partners: an alpha-proteobacteria and an archaeal cell from the TACK/Asgard family - suggesting the involvement of a community of partners. In fact, the most widely cited models are very similar when compared to the 3rd space model and differ only in the path to the eukaryote cell, not in the changes in topology that underlie eukaryogenesis.*

Author’s Comment: The model is *about* the *pathways* to a eukaryotic cell from a starting topology of prokaryotic primary spaces to a more complex topology. It identifies (not necessarily exhaustively) three different classes of potential pathway and focuses on one of these classes (the third space model). Each model begins with two types of prokaryotic space and ends with a eukaryotic-like structure with three secondary spaces (mitochondria, cytosol, nucleus). Other classes of topological transformation are possible, for example starting with only one primary space, or four, five or six etc. primary spaces. These are not considered here, essentially because the literature strongly supports at least two spaces, the archaeal and bacterial starting spaces. It also keeps the model manageable. The reason for exploring the third-space class of models further is that there are some versions of it that allow eukaryotic cells to evolve without requiring phagocytosis or endosymbiosis, a prerequisite feature of most (admittedly not all) other current models of eukaryotic origins.

*Moreover, the author does not do enough to make the case that the eukaryotic genome is a patchwork that is likely to have arisen as the result of DNA influx from a large number of partners. The model proposes that the extracellular space in which the archaeal and bacterial partners that gave rise to the eukaryotic cell are growing when they enter into a symbiotic relationship, becomes the cytoplasm. In a process that has not been discussed in any detail this then becomes bounded by the plasma membrane.*

Author’s comment. The formation of the cytosol was discussed as “matrix replacement” in the original version of the text, but I agree it was much too brief and not developed. A new section has been added to address this issue. *“Membrane encapsulation and cytosolic takeover”* (particularly paragraphs two and three of this section). This is discussed further below.

*Finally, the author suggests that this interpretation of events explains many features of eukaryotic cell organization. While this view is novel, and is an entirely new take on the topology changes required to give rise to the eukaryotic cell, the author has not done enough to lay out the topology of the eukaryotic cell and topological changes required to generate it.*

Author’s comment. The text has been edited to expand on these issues. The development of the topology of the cell is given in several places but particularly section. *“Assembly of a primitive nucleus”* (for the nucleus)*,* and *4.2. “Membrane encapsulation and cytosolic takeover”* (for plasma membrane and cytosol) and *4.6 “Membrane-enveloped organelles”.* (perinuclear space, the endoplasm reticulum, Golgi lumen and the lysosome).

I have, in addition, edited section 1 that lays out the “rules” of the topology. This clarifies (hopefully) that spaces are functionally defined, can communicate, and what the difference is between secondary and tertiary spaces.

*The annotation used here is problematic rather than helpful, in that it allows the author to suggest simple rules that make little physical sense.*

Author’s comment: I kept the original annotation in section *“Three distinct pathways that may lead to the tripartite eukaryotic topology”;* but removed it from the rest of the manuscript as it could prove confusing when used in those more complex descriptions. I appreciate that the annotation is not a mainstream approach to biological description, but I believe that that presenting the transitions in a highly simplified and abstract form is, nevertheless, useful as it strips away the “fog” that surrounds such questions.

*For example: i) The nucleus and cytoplasm are treated as distinct compartments in the model. However, they are not topologically distinct. In every cell cycle they transiently become one during mitosis. In interphase are only separated by nuclear pores, which regulate traffic, not by a membrane barrier.*

Author’s comment. Regarding the dissolution of the barrier between the nucleus and cytosol in interphase, it depends on whether the organisms in question display “open” or “closed” mitosis, both forms being well documented. This question worried me as well, which is why I suggested that the model results in a closed rather than open form of mitosis (paragraph beginning “Mitosis is highly variable”). Taken together, the different forms of closed mitosis have a very wide occurrence. When mitosis is open, the reviewer is correct, the tripartite topology ‘collapses’ to a transient bipartitite state. This now inserted into the text. See section *“Assembly of a primitive nucleus”,* from “If nuclear division originated from fission of L-form-like multi-genomes…” to line 559 “…considered to have transiently devolved to a bipartite state.”

The biology of “open” vs “closed” mitosis has been well reviewed (Raikov, European Journal or Protistology, 1994, *The diversity of forms of mitosis in protozoa: a comparative review*, 30, 253–269). In metazoans, the nuclear membrane breaks down, and mitosis is therefore said to be open. When the nuclear envelope is retained, however, mitosis is said to be closed. There are further variants in which the nuclear membrane is partially retained (semi-open mitosis). The manuscript predicts that the first mitosis was probably closed. In addition to membrane status mitosis is further categorized based on the position of the spindle poles. In orthomitosis the spindle poles are at right angles to the plane of mitosis (as in animal cells), but in pleuromitosis the poles are off-angle to the plane. Closed orthomitosis occurs in various fungi, diatoms and ciliates. Pleuromitosis happens only in closed or semi-open mitosis. Closed intranuclear pleuromitosis, occurs in various fungi and foraminifera. In a further variation, the spindle may even be outside the nuclear envelope and attaches to chromosomes by “insertion points” at the nuclear membrane itself. This is called extranuclear pleuromitosis, and is found in dinoflagellates and trichomonads. Taken together, therefore, the different forms of closed mitosis have a very wide occurrence.

The connections between spaces, including the cytosol and nucleus during interphase, are covered in Section “*Minimal Cellular topologies”*. The secondary spaces are specifically defined as functional, and are “delineated” or “delimited”, which is not quite the same meaning as “surrounded”. There is no reason why they should not be in communication, provided the communication is “gated” in some fashion (Lines 178 to 18 starting “*2. The secondary topology reduces cells to secondary spaces or domains”*. The issue of nuclear pores was discussed in *4.6 Membrane-enveloped organelle"*.

*ii) There are good arguments for considering the perinuclear space, the ER lumen, Golgi lumen and the lysosome as topologically equivalent to the outside of the cell. For example, material fuses to the plasma membrane.*

Author’s comments. This is a very important point, and I agree with it to a considerable extent. Section “*Membrane-enveloped organelles”* has, therefore, been edited to acknowledge the connection outside the cell, and hopefully clarify it with respect to the proposed model. I am not sure, however, that the lumens of these organelles are topologically outside the cell in quite the same way that the lumen of the gut, for example, is a complete topological extension of the outer world. To mark this, I now refer to this compartment as a “topological interface”. In the proposed model, these compartments are part of the tertiary topology (as outlined in section 1, “*Minimal Cellular topologies”*).

*iii) The cytoplasm of a eukaryotic cell is not very different to the cytoplasm of an archaeal/bacterial cell. What is the evidence to suggest it is a new structure derived from extracellular material? iv) In the model it is not clear how the plasma membrane arises and how is it maintained so that it grows and divides with the rest of the cell. In summary, the author has proposed a new topological model. Such a model must explain the topology of the eukaryotic cell in detail and explain how it arose.*

Author’s comment. Points iii and iv. I agree that this was not clear in the original manuscript. It was covered briefly near the end, but in retrospect, this was inadequate. A new sub-section has been added (*“Membrane encapsulation and cytosolic takeover”*) that discusses both the formation of a plasma membrane and the generation of the cytosol. In the original, the transformation of the extracellular matrix to a more cytosolic structure was referred to as *matrix-replacement*. To better emphasize that this is the process whereby the third space becomes transformed to the cytosol, this is now called “*cytosolic-takeover”*. Briefly, the matrix is replaced by cytosolic components that originate from within the resident cells of the matrix ensemble. This occurs as a development and extension of excretory process that already exist within prokaryotes. The matrix undergoes a series of changes, passing from a purely extracellular polysaccharide-based substance, to a transitional state that is partly polysaccharide-based matrix and partly cytosolic (protein), increasing in cytosolic content as it approaches the final eukaryote like state. In this view, it is inevitable that the cytosol and nucleoplasm would be related to the cytoplasm of an archaeal/bacterial cell.

**Reviewer recommendations to authors**

*The author needs to make the case for true chimeric ancestry when there are many other reasons why gene trees might point to multiple prokaryotic contributors to eukaryotes. In particular, the challenges of reconstructing relationships among such ancient sequences is fraught with challenges and artifacts (changes in base composition, long-branch attraction, and convergent selection) that can readily distort the phylogenetic trees. And this is further compounded by the HGT among bacterial lineages and possibly between bacteria and early eukaryotes.*

Author’s comment. I agree we cannot be confident about very distant phylogenetic relationships, for the reasons mentioned. This is now noted in the text from Paragraph 4 of the Introduction “Recovering the detailed relationships among very ancient genomes is profoundly challenging with many opportunities for artifacts and error. Nevertheless, some general conclusions can be made, among them that eukaryotic genomes are mosaics of bacterial-derived, archaeal-derived and eukaryotic-specific genes.”

Simply bringing two cell types together seems unlikely to generate the degree of mosaicism found in the eukaryotic genome. HGT has, therefore, usually been invoked in order to explain extreme eukaryotic mosaicism. Large scale HGT did, indeed, occur between Bacteria and as outlined in section *“Genomic reorganization towards a eukaryotic pattern”* first paragraph starting “The horizontal transfer of genes………..” . As far as I am aware, however, there is no example of massive HGT transforming an Archaea or a Bacteria into anything other than a modified Archaea or a modified Bacteria (section *“Genomic reorganization towards a eukaryotic pattern”,* paragraph one to three). This is, I believe, a critical question. Conventionally, prokaryotic endosymbiosis is given as the rationale for the transition to a more complex cell type, but it remains unclear how likely, or not, such an event would be, and other possibilities should at least be considered. I have added new text to discuss this in section *“Genomic reorganization towards a eukaryotic pattern”,* paragraph 5: “Although massive horizontal gene transfer did occur between Bacteria and Archaea, there is no example, as far as I am aware, of massive horizontal gene transfer transforming an archaeal species or a bacterial into anything other than modified Archaea or a modified Bacteria. Something else is therefore required in addition to horizontal gene transfer. Prokaryotic endosymbiosis has been proposed as one such driver, but given its rarity in nature, other possibilities should be explored*.*”

Communal living is the dominant lifestyle of most prokaryotes (*Flemming HC, Wuertz S. Bacteria and archaea on Earth and their abundance in biofilms. Nat Rev Microbiol. 2019)*. The suggestion made here is that living communally first super-charges HGT (this is supported by experiments) and second, that a transition to cell wall-free cells, protected within the matrix/proxy cytosol, allows increased cell fusion (a relic of which is the fusion/fission cycle of extant mitochondria) and this further amplifies the exchange of genetic material until structures (called here multi-genomes) emerge that are neither definitively Archaea or Bacteria. The reorganization of the multi-genomes within the proxy cytosol tends towards a eukaryote-like life form.

*Second, the model is not viable without a clear notion of a cellular “space.” In what sense are the cytoplasm and nuclear lumen different spaces when there is no membrane boundary between them?*

Author’s comment. Please see above, for a discussion of closed and open mitosis.

*And what space or spaces do the ER lumen and perinuclear space belong in the model?*

Author’s comment. They are tertiary spaces, as they derive from either in-folding of the plasma membrane, or out-folding/budding of the primitive nuclear membrane. Please see section “*Membrane-enveloped organelles*” for discussion of this point.

*In short the author needs to base the notion of space in modern eukaryotic cell biology terms.*

*Third, the model needs to explain the origins of the eukaryotic cytoplasm and plasma membrane.*

Author’s comment. Please see above for discussion of this point.

*Is the author suggesting that the archaeal partner pumped ribosomes across its plasma membrane to fill this space?*

Author’s comment There are several ways that the ribosomes could pass from the resident cells to the transitional matrix/proxy cytosol. I favour cell lysis. If a prokaryote can ‘pump out” bacteriophage by regulated autolysis I don’t see why a similar mechanism cannot be co-opted to pump out any other structure. This has been edited and is in the paragraph starting “Cell lysis, vesicle budding, or leakage across an L-form plasma membrane in section *“Partitioning of protein synthesis to the cytoplasm”*. Altruistic programmed cell death/cell lysis has been postulated in biofilms, and is equally applicable in a third-space model. It is one feature of the proposed model that definitively requires the presence of a cell population as programmed cell death and lysis of a population of one would halt the process dead in its tracks.

*How did the plasma membrane come to be?*

Author’s comment. Please see paragraph one of section “*Membrane encapsulation and cytosolic takeover”* and the discussion above.

In light of these major flaws, we believe that the model proposed in this paper is not appropriate for publication in its current form.

### Reviewer’s report 3: Michael W. Gray, Affiliation Department of Biochemistry and Molecular Biology, Dalhousie University, Canada.

**Reviewer comments to Authors.**

*I did not review the original submission of this manuscript, which has since been comprehensively revised to take account of the very extensive comments and criticisms of the two reviewers who did see the original. Considering the in-depth review the manuscript has already received and the resulting changes made by the author, my comments are necessarily limited. The subject of this submission is undoubtedly of considerable interest. As the author points out, none of the numerous extant models of eukaryogenesis compellingly accounts for the all of the known properties of the eukaryotic cell and its genomes (nuclear and mitochondrial). For that reason, new ideas are always welcome as a means of advancing discussion of this challenging problem. The author’s model in which a biofilm-like matrix might have been central to initiating the process of integrating separate bacterial and archaeal partners is an interesting one and, in principle, deserving of publication. The author has argued his case well and, in general, the presentation flows smoothly. I did find parts of the text heavy going, in part because my own knowledge of the biofilm literature is limited. On the other hand, the figures illustrating the main points of the model are quite helpful (but see Minor issues, below). In general, I did not find the overall model particularly persuasive in a number of its aspects. In particular, the biofilm-to-cytoplasmic membrane transition is, in my view, difficult to imagine.*

Author’s comment. I agree it is difficult to imagine. The third-space model is obviously speculative, and is intended as a first pass at developing such a model, but it is based on the normal mode of life of prokaryotes (in communities) and on processes that can be observed in the wild or in laboratory conditions.

The model discussed in detail is the “strong” version, but, as outlined in section “Strong, weak and intermediate third-space models”, there are other versions, (“weak” and “intermediate”). I chose to focus on the strong version with a mixed population in order to see how far the idea could be pushed. It requires a shift in emphasis from prokaryotic endosymbiosis, or other form of cellular engulfment, which have been rarely observed, and in which only two cell types are the primary players, to an exosymbiotic process which can include more than two cell types. Everything in the model arises by small steps. Originally, I used the term “progressive” for this, but to emphasize this feature I have, in places, switched to the term “incremental” which hopefully better captures the meaning. This avoids unexplained evolutionary leaps across structural and functional chasms. The formation of the boundary membrane is a key step, and this may be hard to envisage. Additional material was, therefore, added (second paragraph of * “Membrane encapsulation and cytosolic takeover”*) to outline three possible ways a boundary membrane could form around a cellular ensemble in a matrix. The first paragraph of this section outlines an observed example of a bacterial colony that is, at least under some conditions, enclosed within an apparent lipidic membrane-like sheath. In addition, it points out that lipid substrates for the formation of the membrane are likely to be available in the matrix.

Once contained within a boundary membrane, the lysis of a subset of cells in the population and/or regulated secretion from the live cells of the population (both process have been observed) would deposit cytoplasmic material into the matrix. Given time, and in a population of cells linked by obligatory exosymbiosis (symbiosis and syntrophy between cells is often observed), these processes initiate the progressive replacement of the matrix by the cytoplasm. Again, replacement of the matrix by the cytosol would not occur as a step function but as an incremental process. As the ensemble transitions towards a more cell-like lifestyle, the production of the incipient cytosol becomes a more regulated and orderly process. A summary is now added in the final paragraph of section “*Membrane encapsulation and cytosolic takeover*”.

*Also, in contrast to endosymbiotic models, the three-spaces model does not readily account for the selection of an archaeal cell as the progenitor of the nucleus and a bacterial cell as the forerunner of the mitochondrion.*

Author’s comment. The strong-third space model hypothesizes that cells in the matrix-ensemble undergo genetic merging (to become the so called multi-genomes). Extensive gene transfer in both directions between Arcahea and Bacteria has been documented. The multi-genome structures contain both archaeal and bacterial genetic material. This is exactly what is seen in the eukaryotic nucleus, that is, a genome with contributions from both prokaryotic domains. It is from these structure that the nuclei develop. To emphasize the mixed nature of the nuclear precursors in the third space model I have referred to them as bacteriarcheons. The third space model assumes that systems were selected because they were “fit for purpose” (last sentence, section "*Steps towards a eukaryotic cytosol and mitochondria: shared metabolism *") and not because they were specifically archaeal or bacterial. This is re-stated in section “*Limitations*” part 2. In this view archaeal information processing provided an advantage over the bacterial machinery. Once one part of the archaeal information processing machinery was selected, other systems that interact with it would be brought along in-step in order to maintain the biochemical and functional compatibility of the systems. Once a critical archaeal information processing system was selected, the evolution of supporting nuclear functions would, therefore, be channeled along an archaeal line. The concept of associated mechanisms being carried along by functional linkage is discussed in the final paragraph of *Partitioning of protein synthesis to the cytoplasm* and part 2 of *"Limitations".*

The selection of mitochondrial precursors is discussed in *“Steps towards …. Shared metabolism”,* and required an aerobic, mesophilic cell capable of generating enough ATP to provide a surplus. Clearly some member of the alpha-proteobacteria must have fit the bill.

*Moreover, why the bacterial partner would selectively undergo reductive genome evolution, ceding many genes to the archaeal partner, is also not well developed. Again, endosymbiotic models better account for this distinction (it is well established,* e.g.*, that the bacterial partner in obligate endosymbiosis generally has a much shrunken genome compared to its free-living relatives).*

Author’s comment. I have now added a short discussion of this problem in text “Moreover, as the mitochondrial precursors …… mosaicism of the mitochondrial proteome”. Interestingly, exosymbiotic prokaryotic cells can also show genome reduction [192], so the phenomenon is not restricted to endosymbionts.

*These and other concerns aside, the model presented here is still worth considering and, if possible, refining.*

**Minor comments.**

*1.The author uses the terms “eukaryotization” and “eukaryogenesis” seemingly interchangeably throughout the manuscript. Are these terms equivalent in the author’s view? If yes, perhaps only the more common “eukaryogenesis” could be adopted to avoid possible confusion. If no, the terms should be explicitly defined in order to clarify their distinction.*

Author’s comment. This has been altered to eukaryogenesis throughout

*2.L68–70 (ref,. 27): Additional references linking intracytoplasmic membranes in alphaproteobacteria to mitochondrial cristae could be added: DOI:* 10.1093/molbev/msw298*and DOI:* 10.1016/j.cub.2015.04.006*.*

Author’s comment. These references have been added

*3. L407: Bacteria (cap.) 4. L414–415: should read “an archaeal or a bacterial species” 5. L642: coli 6. L644: vesicle-associated 7. L724: membrane-enclosed 8. L1451: delete “34.” 9. L1812: “243 of bacterial origin” in Fig. 1(A), the number is 234. Also, Fig. 1(A) retains “eubacterial” (rather than “bacterial”) and “eub/arch” (which presumably should be “bact/arch”*

Author’s comments. 3–9. Each of these points have been corrected.

*10. I find Fig. 1(A) confusing in that the figure legend states, “Among the bacterial clades, 41 were clearly alphaproteobacterial ...”. This implies that the 41 shown in the figure (and the 198 non-...) are actually contained in the 234/243 bacterial group to the left, rather than being separate. It would help if the figure could be revised in some way to make this point explicit.*

Author’s comment. This has now stated in the legend.

*11. L1815–1816: “Only 3 Clades ...” The meaning of this sentence is unclear. Does the author mean that the clades in question do not branch with either Bacteria or Archaea? In which case, why are these clades included in the bacterial group (or are they?).*

Author’s comment. This paper this was based on is rather complex, and I have used only one level of their analyses. I rewrote the sentence “only 3 clades …” which now reads as “Trees were generated for eukaryotic, bacterial and archaeal gene families. These were then analyzed in terms of “configurations”, for example, those that branched cleanly between eukaryote and bacteria, were assigned as bacterial clades, etc. Only 3 clades (labelled bact/arch) have the so-called “three domain configuration”, that branched into Archaea, Bacteria, and Eukarya with no obvious bias between the three domains.”

### Reviewer’s report 4: Damien Devos, Affiliation European Molecular Biology Laboratory (EMBL), Heidelberg)

Reviewer two-second version

*The author has convincently addressed most of my concerns. I would now recommend the article for publication. All considered, it is an hypothesis as worthy of publication as any other. I do have however, I few minor concerns to consider: Answer to my concern with L45. I was asking about clarification of the age of the archaea in the sentence, “making them much younger than Archaea or Eubacteria”. Changing the sentence to “making them much younger than prokaryotes” is not changing anything at all. This doesn’t clarify anything about the age of archaea. I do however agree that the article is long enough to discourage a more profound discussion about the age of the archaea. Despite the author’s understanding that “eubacteria” is an inadequate historical term and as corrected in the text, fig 1 still refers to “eubacterial”, “eub/arch”,*

Author comment. This has been corrected

*… Ref* 54 *is about a prepublication deposited in bioRxiv. I am unsure about this journal’s policy concerning preprints.*

Author comment. A more complete article has now appeared and is cited (reference (58)).

*The new paragraph “Membrane encapsulation and cytosolic takeover” is still not satisfying to me, as it briefly provides potential lipid sources, not an explanation of how these would have formed around the third space. How would the third space be transferred internal to the lipids?The Myxococcus example is unclear, to say the least.*

Author comment. The section is now re-written. I have added additional text that expands upon three possible pathways for membrane formation (paragraph 2 of *Membrane encapsulation and cytosolic takeover)*. Th first paragraph of this section discusses extracellular lipid sources in a matrix, and a documented case that these lipids can be assembled into a sheath-like outer boundary.

Returning to the Myxococcus example, the observed existence of a membrane-like structure around the Myxococcus colonies shows that under some circumstances some bacterial communities can enclose themselves in a membrane-like structure. It is certainly true, however, that Myxococcus is not representative of typical biofilms as the colonies are motile and predatory. New text “Although Myxococcus, being motile and predatory may not be representative” was added to outline this (paragraph one 4.2. “*Membrane encapsulation and cytosolic takeover*”).

*About our question on the assumption that eubacteria outnumbered archaea in the matrix to justify eubacterialization of the archaeal component, as a posteriory thinking, author response is not convincing and the point is not addressed in the section “Genomic reorganization towards a eukaryotic pattern”.*

Author comment. The model does not require more bacterial and archaeal cell types, it can accommodate only two cell types, although I think there are advantages to a mixed population with more than two bacterial cell types. This was outlined in the previous version of the text i.e. “In its simplest version, the third-space model requires only two cell types, or a population of two cell-types, embedded in the matrix, one of which must be archaeal and the other bacterial.”

Allowing for mixed populations in the matrix, the phylogenetic makeup of the eukaryotic genome (Fig. 1) suggests that it arose from more than one bacterial genetic reservoir but does not require multiple archaeal reservoirs. The third-space model accounts for this by allowing for a population of different bacterial cells in the matrix ensemble. New text has been included in the first paragraph of section “Genomic reorganization towards a eukaryotic pattern” to include the following

"The eukaryotic genome arose by merger of archaeal and bacterial genomes. The third-space model can accommodate simple populations of only two cell types (an archaeon and an alpha-proteobacteria) but also more complex mixed populations with more than two cell types. Considering mixed populations, and focusing on the bacterial component, the relative weakness of the alpha-proteobacterial signal (the mitochondrial precursor) compared to the aggregate bacterial signal in the eukaryotic nuclear genome (Fig. 1) can be rationalized if the population contained more than one type of bacteria (an alpha-proteobacterium plus others). A mixed population model does not, however, necessarily require the involvement of more than one archaeal cell type. The minimal mixed-population third-space model suggests, therefore, more than one type of bacterial cell interacting with one archaeal cell type (presumably one of the Lokiarchaeota).

## Data Availability

Not applicable.
